# A micro X-ray computed tomography dataset of fossil echinoderms in an ancient obrution bed: a robust method for taphonomic and palaeoecologic analyses

**DOI:** 10.1093/gigascience/giy156

**Published:** 2018-12-07

**Authors:** Mhairi Reid, Emese M Bordy, Wendy L Taylor, Stephan G le Roux, Anton du Plessis

**Affiliations:** 1 Department of Geological Sciences, University of Cape Town, University Avenue, Upper Campus, Rondebosch, 7701, Cape Town, South Africa; 2 CT Scanner Facility, Central Analytical Facilities, Stellenbosch University, Private bag X1, Matieland, 7602, Stellenbosch, South Africa

**Keywords:** micro-CT, µCT, 3D imaging, virtual taphonomy, obrution deposit, echinoderms

## Abstract

**Background:**

Taphonomic and palaeoecologic studies of obrution beds often employ conventional methods of investigation such as physical removal and extraction of fossils from their host rock (matrix) by mechanical preparation. This often-destructive method is not suitable for studying mold fossils, which are voids left in host rocks due to dissolution of the original organism in post-depositional processes.

**Findings:**

Microcomputed tomography (µCT) scan data of 24 fossiliferous rock samples revealed thousands of Paleozoic echinoderms. Digitally "stitching" together individually µCT scanned rock samples within three-dimensional (3D) space allows for quantifiable taphonomic data on a fossil echinoderm-rich obrution deposit from the Devonian (Emsian) of South Africa. Here, we provide a brief step-by-step guide on creating, segmenting, and ultimately combining sections of richly fossiliferous beds to create virtual models suited for the quantitative and qualitative taphonomic analyses of fossil invertebrate assemblages.

**Conclusions:**

Visualizing the internal features of fossiliferous beds in 3D is an invaluable taphonomic tool for analyzing delicate fossils, accounting for all specimens irrespective of their preservation stages and with minimal damage. This technique is particularly useful for analyzing fossiliferous deposits with mold fossils that prove to be difficult to study with traditional methods, because the method relies on the large density contrast between the mold and host rock.

## Data Description

### Motivation and background

Microcomputed tomography (micro-CT or µCT) and three-dimensional (3D) visualization techniques have become an increasingly popular tool used in many fields of paleontological research [1–4], especially in anatomy and functional morphology of vertebrates [5–9] and invertebrates [10–12] and even in micropaleontology [13]. The advantage of this imaging technique lies in its power to construct high-resolution, cross-sectional views of fossils without causing damage during extraction from the rock matrix [13, 14]. Conventionally, paleontologists use mechanical preparation techniques (e.g., air abrasive tools, pneumatic tools) that often damage delicate fossil structures and seldom allow entire specimens to be completely exposed [1]. Moreover, many fossils are preserved as molds where the fossil itself was dissolved away by post-depositional processes leaving only a void in the host rock. Anatomical details are captured only in the impressions of the external surfaces of the original fossil. Advancements in X-ray tomographic technology and data processing software (e.g., VG Studio Max and SPIERS) enable scientists to not only visualize two-dimensional (2D) dissections of scanned fossil material but also to reconstruct high-resolution 3D models of body fossils as well as mold fossils from a variety of host rocks [2]. In recent years, X-ray-based methods have been extensively applied in the morphological analysis of both macro- to micro-size invertebrate fossils [15–17]. The study of fossil echinoderms, a group of marine invertebrates characterized by delicate, multielement calcareous skeletons, has particularly benefited from the use of µCT techniques.

This dataset was created with the purpose of visualizing a complex fossiliferous obrution deposit, focusing on two types of seafloor-dwelling fossil echinoderms found in this rock layer: ophiuroids, or brittle stars, with many modern relatives that are common in oceans today, and an extinct group known as stylophorans. Obrution beds form during storms due to the sudden smothering of the seafloor-dwelling communities (marine benthos) by rapidly deposited storm sediments (tempestites) [18, 19]. By providing snapshots into the paleoecology of marine organisms, obrution deposits often display not only exceptional fossil preservation but also rare behavioral information that would otherwise be lost from the fossil record through destructive taphonomic (fossilization) processes (e.g., decay, disarticulation, fragmentation, transport, scavenging) [20]. Here, we introduce the utility of virtual reconstructions as a means of investigating often complex fossiliferous obrution deposits, with focus on taphonomic assessments of the fossil community rather than investigation of anatomical structures of individual fossils themselves. This µCT technique allowed the 3D visualization of not only the degree of articulation for each individual specimen but also revealed different taxa and relationship to other taxon present within the bed. Furthermore, the imaging of very small (1–3 mm) stylophorans that would have been missed with conventional study was only possible using this high-resolution scanning method. Taphonomic observations such as orientation (oral side up, down, or oblique) of the fossils within the bed, posture, and arrangement of ophiuroid arms and their relative spatial arrangement to one another could all be quantified *in situ*. Here, we also provide a brief step-by-step guide on creating, segmenting, and ultimately combining sections of a fossiliferous layer that contains abundant remains of delicate ophiuroids and stylophorans in order to create a virtual view suited for quantitative and qualitative taphonomic analyses. An accompanying publication [21] presents the results of this analysis.

### Material and methods

#### Excavation of the fossil bed

**Figure 1:**
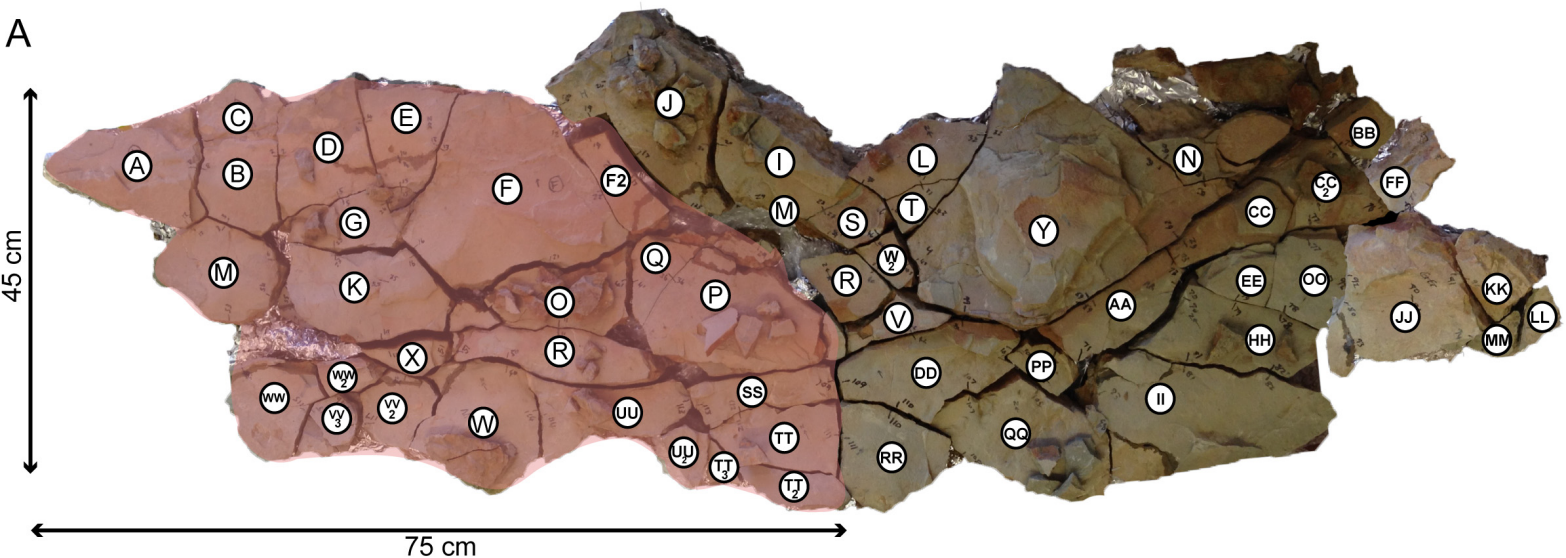
Karbonaatjies obrution bed that was excavated from Karbonaatjies farm (∼145 km northeast of Cape Town, South Africa). Each individual rock slab was given a reference letter in the field and subsequently reassembled in the lab.

The initial discovery of the Karbonaatjies bed occurred during a preliminary study in 2014, when collected samples revealed rare, well-preserved individuals of an undescribed taxon of ophiuroid and stylophorans [22]. The study area is located within the Cape Fold Belt, and the exposed rocks lithostratigraphically belong to the Lower Devonian (Emsian ∼400 Ma) Voorstehoek Formation, Bokkeveld Group in South Africa [23]. The obrution bed was excavated from a road cutting on Karbonaatjies farm, which is situated ∼145 km northeast of Cape Town (GPS: 33°24003.600S, 19°52042.700E). A section of the obrution bed, approximately 2 × 1 m wide with an average thickness of 4 cm, was systematically excavated using a flat brick chisel, geological hammer, and pickax. The highly weathered nature of the outcrop caused the obrution bed to break up into 55 pieces during removal; each piece was labeled from A to WW (Fig. 1). All pieces were carefully reassembled in the lab and photographed.

Conventional paleontological analysis of this deposit posed problems due to the fragile state of the fossils caused by the deep chemical weathering of the originally calcareous fossils, leaving only voids in the host rock, which is a silty, very fine-grained sandstone. For this reason, µCT scanning was used to analyze the Karbonaatjies obrution bed, which showed, among others things, that the sampled portion of this 2- to 3-cm-thick layer contained more than 1,000 ophiuroid specimens of a new genus and species and hundreds of stylophorans.

#### Scanning, data processing, and quality control

The µCT scanning was performed at the Stellenbosch University Central Analytical Facility with the aid of a walk-in microfocus X-ray CT scanner (General Electric Phoenix V|Tome|X L24 model with additional NF180 option [24]). The CAF micro-CT instrument has a typical minimum voxel size of between 1 and 100 μm and can be used for samples that are up to 300 mm long and 200 mm wide. Samples were placed within a plastic bottle supported by dense polystyrene foam for scanning. Typical considerations for scan setup and parameter choices are outlined by du Plessis et al. [24]. A small wax ball was stuck to the upper surface of each sample in order to indicate right-way-up orientation as well as the relative position to other samples within the bed. To ensure that the X-ray spot size did not exceed the selected scan resolution, optimal X-ray scan parameters were chosen while using live digital X-ray images (e.g., for ideal X-ray penetration, we monitored the high transmitted brightness values). X-ray settings ranged for voltage from 160 to 240 kV (for larger samples) and for current from 200 to 220 μA depending on sample size, respectively. Detector shift was activated and background calibration was performed before each scan in order to minimize ring artifacts and achieve high image contrast. To reduce potential beam hardening artifacts, a 0.1 mm copper beam filter was used in all the scans. The samples in this study are relatively dense, rigid sandstone slabs with an average length of 200 mm. To obtain sharp images of larger samples, the voltage had to be raised up to 240 kV on the high-power tube allowing for more beam filtration (less beam hardening) and limiting the generation of other artifacts [25]. Scan time averaged from approximately 40 to 60 minutes depending on the size of the sample. Longer samples were scanned in sections (allowing for higher magnification). Using an exposure time of 500 ms per image, images were acquired in steps during a full 360° rotation. At each step position, the first image was discarded and the next two images averaged to obtain lower noise and sharper images. The acquired projection images (between 1,400 and 2,600 images per scan) were reconstructed using system-supplied Datos reconstruction software, where the choice of numbers of projections depends on sample size and magnification and was selected according to the guidelines in du Plessis et al. [24].

#### Digital analysis

The tomographic reconstruction dataset obtained from scanning was visualized and analyzed using the VGStudioMax 3.1 software package (website: [26]) to create a 3D view of individual fossils within each sample. This software was also used to produce images (e.g., screenshots) and animations.

**Figure 2:**
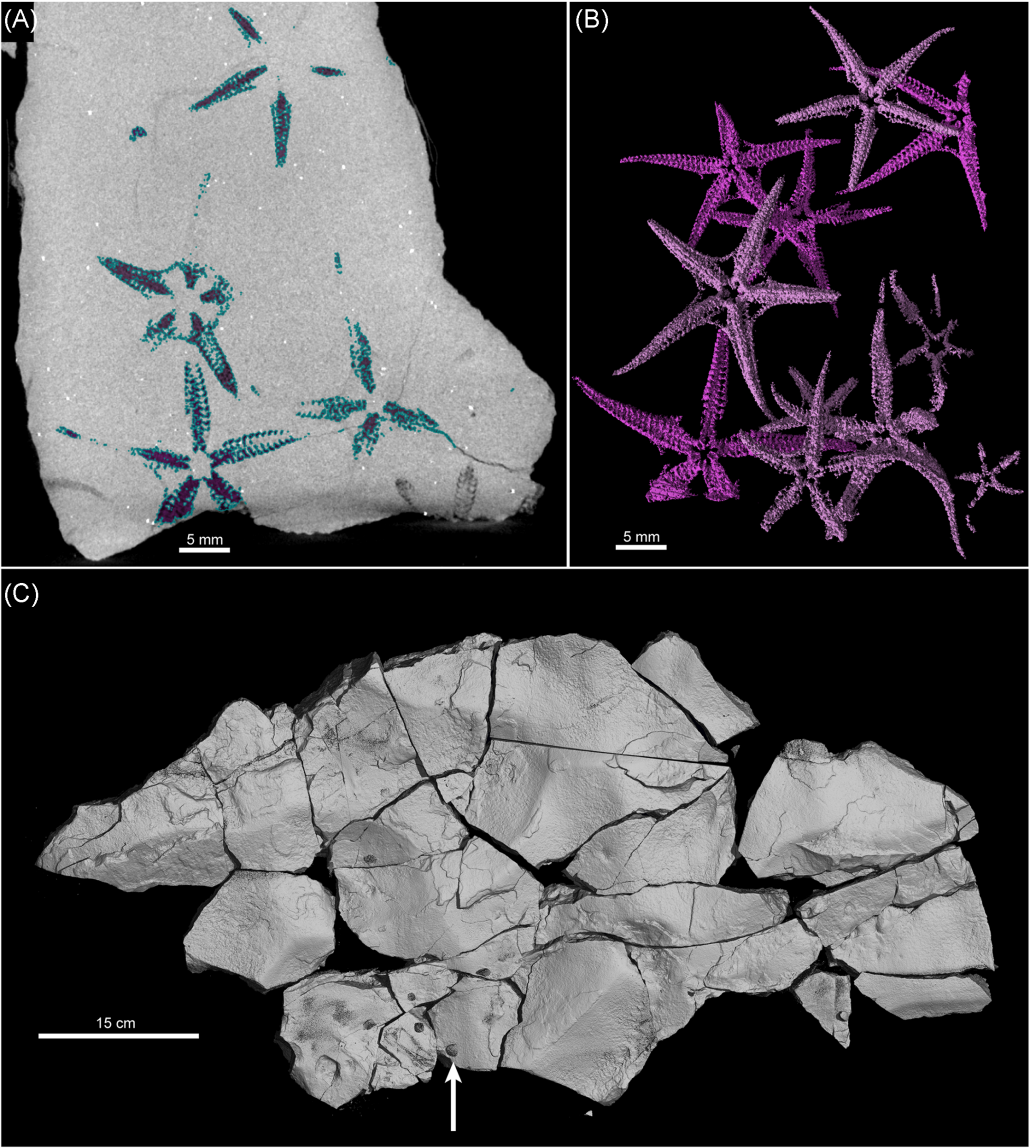
**(A)** Sample II, a long sample (26 cm x 12 cm) illustrating beam hardening artifacts causing the center of the sample to appear to have darker voxels while the edges appear much brighter, even though the sample is homogeneous. **(B)** Side view of sample II showing how the beam hardening artifact causes the pyrite minerals to give a "starburst" appearance. **(C)** Sample II after being cut and rescanned following the above-mentioned procedure.

Virtual preparation and dissection of the specimens involved a series of modified steps outlined by Abel [27] and Matthews [28] as follows: (1) Density contrast enhancement: generation of a larger contrast between the gray scale values that represent the rock and surrounding air by optimizing the gray value range on the histogram. (2) Register object: alignment of the sample to a specific coordinate system so that the top-down 2D viewer of the slices scrolls through the sample parallel to the bedding plane. (3) Surface determination: defining the material boundary of interest. This is generally the quickest and easiest way to separate a region of interest (ROI); however, this was not possible because the fossils (preserved mostly as void space) have the same density or gray scale value as the permeating cracks in the samples and the surrounding air. It is for this reason that the region growing tool was predominantly used. (4) Region growing: generating a selection using a region growing algorithm (Fig. 2A). This is one of the simpler image segmentation methods used for 3D data, which essentially establishes the ROI (i.e., subparts of the volume data). The region growing tool allows the selection of a "seed’"point (in this case, the "black" voxel of a fossil or the voids within the rock sample); the algorithm will expand the selection to all voxels connected to that seed point based on a defined tolerance of voxel gray values relative to the selected seed point. The threshold (selection of voxels with gray values within the selected gray value interval) changed from sample to sample but was generally around ±5,000 in this study. Region growing was the most time-consuming step, as each individual fossil had to be segmented out in order to make the 3D volume rendering. Generally, when a fossil is scanned, the minerals that make up the rock and the fossil itself are compositionally differentiated so that there is enough contrast to allow anatomical structures to be digitally visualized in 3D. This highlights one of the difficulties with CT scanning of fossiliferous rock samples. If the compositional difference between the fossils and the host rock (matrix) is negligible, little or no information will be captured in the scans. However, the fossils in this study are predominantly mold fossils (i.e., 3D imprints left behind in the host rock after the original organism was dissolved) with no internal mineralogical information, and this made segmentation much easier in that the contrast between the fossil (now air-filled void) and surrounding rock is very high. (5) Finally, the last step involved volume rendering: generation of a 3D volume from the segmented 2D ROIs using a specific rendering algorithm, in this case, the isosurface render. Isosurfaces are mathematically defined surfaces calculated from a volume along points of interest [3]. The dataset is treated as a volume comprising voxels (3D pixels that contain measurements of color) instead of 2D pixels. Once the volume is created, the appearance of the volume objects can be manipulated (e.g., color, transparency) to visualize the fossils in 3D (Fig. 2B). Last, once the fossils were segmented out, rendered in 3D, and false-colored accordingly, the fossiliferous rock samples were virtually "stitched" together to recreate a section of the obrution bed (Fig. 2C). This was achieved by using the volume import tool; each sample had to be manually aligned and placed in 3D space.

## Data Quality and Limitations

**Figure 3:**
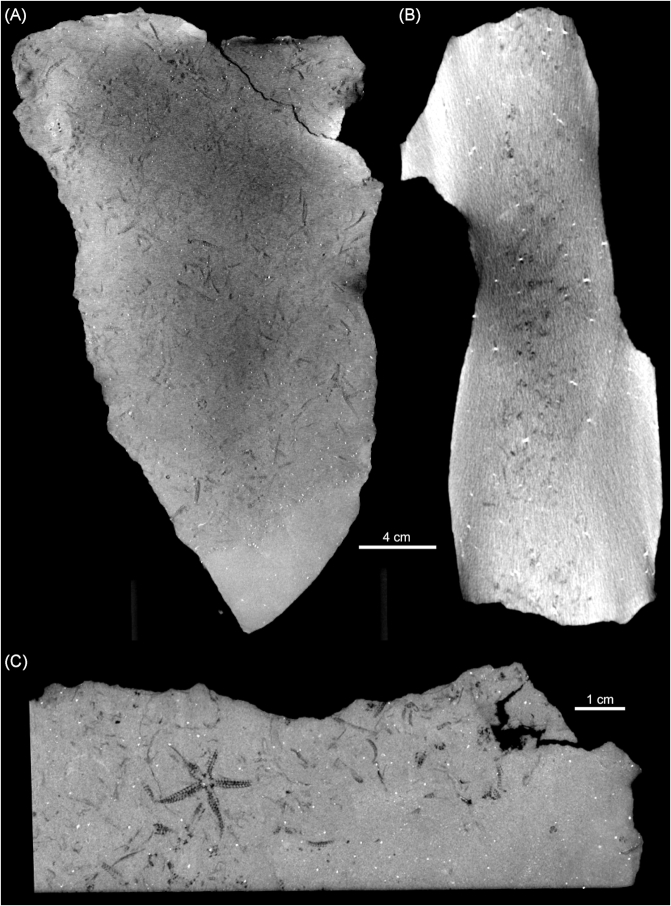
**(A)** Segmentation of ophiuroid specimens using the region growing tool in VGStudioMax. **(B)** The resulting virtually reconstructed ophiuroids in 3D, rendered with color and surrounding matrix set to transparent. **(C)** Reconstruction of a portion of the fossil bed in 3D (shaded area in Fig. 1). Black arrows point toward wax balls stuck to the upper surface of the samples to indicate right way up and relative positions.

In spite of the general preventative measures (e.g., using a copper filter, scanning perpendicular to the long axis of the sample), a number of the larger and longer samples scanned had artifacts that obscured details in the CT images. This makes interpretation and analysis very difficult, sometimes even impossible (Fig. 3A, 3B). The major type of artifact identified is beam hardening, which is a problem that arises when a high-energy polychromatic X-ray source is used to penetrate a dense sample. The strong absorption of the beam in a large sample causes low-energy photons to be absorbed more strongly than high-energy photons, resulting in unequal absorptions and giving rise to this type of artifact. This problem often occurs in paleontological samples because of the high density and large size of the fossil specimens and their matrix, resulting in low transmission and high noise [3]. In our study, it made identification and separation of the echinoderms arduous and even impossible in some cases, as more details toward the center of the samples were lost. To rectify this, many samples were cut into smaller sections and most of the non-fossiliferous host rock matrix was removed (Fig. 3C). These samples were subsequently rescanned in sections to obtain higher magnification, especially for longer samples. Longer samples were scanned using a vertical multiscan procedure whereby different parts were scanned automatically with some overlap; the reconstruction software performed automatic stitching of multiple scans according to the accurate vertical translation distances used (no manual interface is necessary). However, due to the high power required, the X-ray source often became unstable, causing errors and failed scans. For this reason, some individual parts of larger samples were scanned separately and manually stitched. The advantage of this is that if there is a failure, only one part needs to be rescanned and less overlap is required for manual stitching, reducing the number of scans required for long or large objects. Finally, while all 55 samples were CT scanned, time and funding constraints allowed for only half of the bed (24 samples) to be rendered in 3D.

## Potential Uses

**Figure 4:**
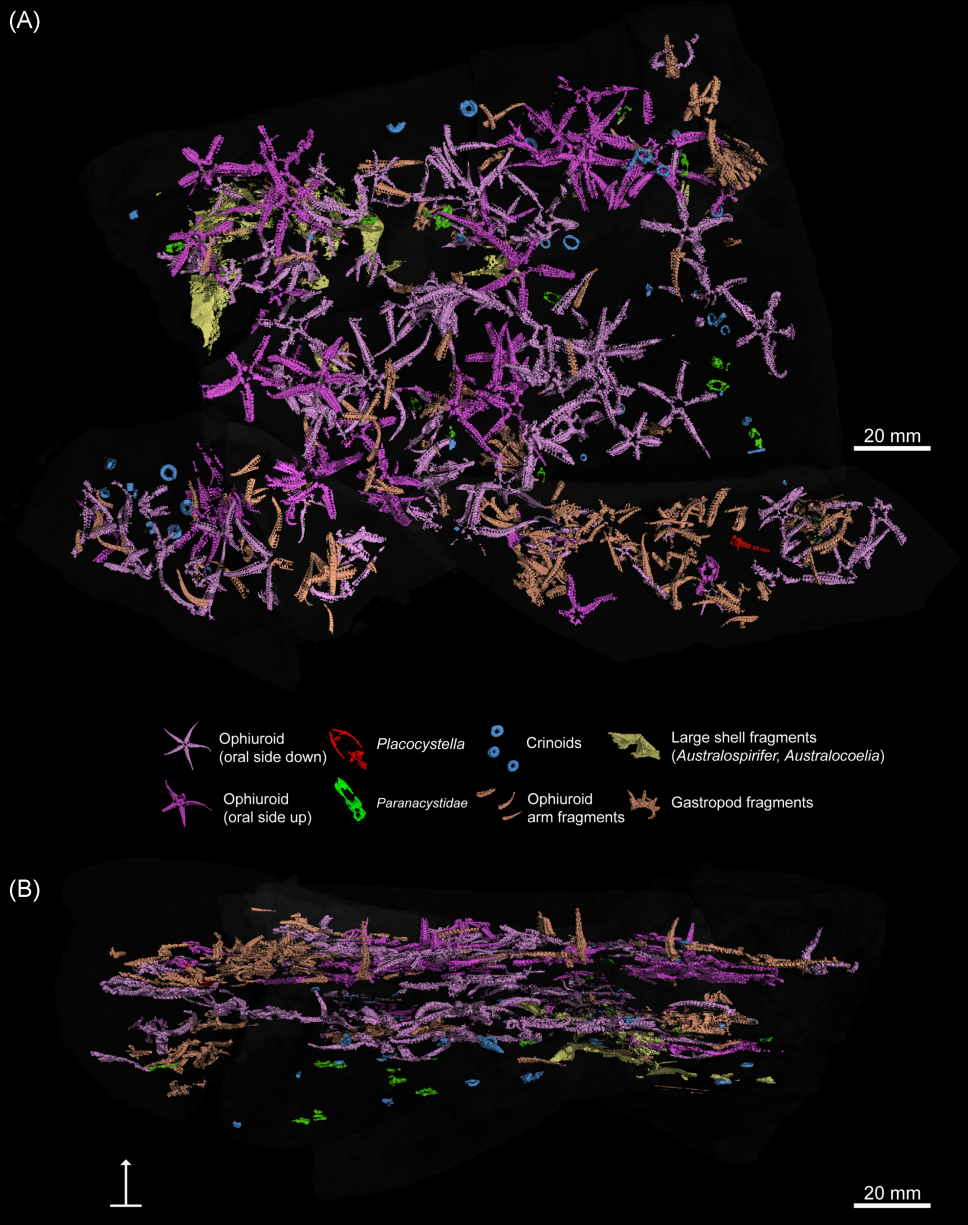
**(A)** Virtual reconstruction of samples SS, TT, TT2, TT3, and UU2 rendered with lights, color, and surrounding matrix set to 90% transparent. Approximately 80 articulated ophiuroids (light pink = ophiuroids oral side down; dark pink = ophiuroids oral side up), 13 paranacystids (green), 1 *Placocystella* (red) aulacophore fragment, numerous crinoid ossicles (blue), fragmented ophiuroid arms (pale orange), and large shell fragments (yellow) are all shown in 3D. **(B)** Side view shows three vaguely defined ophiuroid "horizons" as well as ophiuroid arms extended upward within the obrution bed.

The dataset presented can be used as an example of how taphonomic and paleoecological investigations can be conducted using µCT-scanned rock samples within 3D space. Colorizing the different taxa present in the virtually reconstructed fossiliferous bed played an important role in the taphonomic assessments. Ophiuroids were color-coded according to their different orientations, light pink for oral (mouth) side up and dark pink for oral side down, which are their presumed life position (Fig. 4A). Quantifying the percentage of ophiuroids in life position is a possible indication of the extent to which the ophiuroids may have been transported by storm-induced current and are subsequently reoriented before burial. Using the 3D reconstruction of the bed, most ophiuroids and stylophorans could be assigned to different taphonomic groups or decay stages based on their level of preservation. For example, ophiuroids that have fully articulated arms or stylophorans with a complete theca and aulacophore can be assigned to group 1, which is intact or complete preservation. Other taphonomic categories were used for specimens in more fragmentary stages of preservation. Paleoecological and taxonomic measurements such as specimen counts, ophiuroid disc or body diameter measured from the base of the arm to the opposite interradius, ophiuroid arm length measured in relation to the disc diameter, and stylophoran theca or body width and length, were all measured directly onto the 2D tomographic images using the digital caliper tool in VGStudioMax.

One unique perspective of virtually viewing the obrution deposit in 3D is that it allows the examination of multiple levels of fossils preserved *in situ* within the bed (Fig. 4B). This is of particular interest because it provides insights into how the storm-generated sudden sediment supply smothered the echinoderms that lived on the Devonian seafloor. In this case, the dense assemblage of ophiuroids is arranged into vaguely laminated horizons with associated fossil shell debris. This helps in our understanding of the initial pre-burial storm conditions and the palaeoecologic fidelity of the resulting deposit. By setting the surrounding matrix to transparent in the program, other features such as the different arrangements of ophiuroid arms and the flexure of the stylophorans can be seen within the 3D space. One of the most striking features of the obrution bed was identified using this technique. We observed that many of the ophiuroids had one or more arms extended upward into the overlying sediment. This has been identified as an escape posture and is comparable to modern examples of ophiuroids escaping from a sudden influx of sediment during storms [29]. Evidence of this behavior in ancient ophiuroids is often difficult to interpret and is rarely observable in preserved ancient ophiuroids because traditional methods do not allow for such a comprehensive 3D view of the fossils [30, 31].

The use of micro-CT scanning in paleontological research has grown by leaps and bounds over the past 10 years. This study allowed the recognition of small, cryptic fossil taxa that would have been otherwise missed as well as the observation of key paleontological features that are critical to the interpretation of the deposit.

## Supplementary Material

GIGA-D-18-00323.pdf

GIGA-D-18-00323_R1.pdf

Response_to_Reviewer_Comments_Original_Submission.pdf

Reviewer_1_Report_1_(Original_Submission) -- Chris Armit 9/19/2018 Reviewed

Reviewer_1_Report_1_Revision_1 -- Chris Armit 10/23/2018 Reviewed

Reviewer_2_Report_1_(Original_Submission)_Reject -- Yu Liu 9/24/2018 Reviewed

Reviewer_3_Report_1_(Original_Submission)_Reject -- Michael Bruce Meyer 9/29/2018 Reviewed

## Data Availability

Data are available to download from the *GigaScience* GigaDB repository [32]. The presented dataset is available as 2D X-ray projection images and stacks of reconstructed slice images for each sample scanned and can be viewed in any image viewer program. The final 3D render of the fossil bed is available as a VGL file that can be viewed on the free downloadable mvVGL program at https://www.volumegraphics.com/en/products/myvgl.html and is also available as a STL file that can be read by standard image viewer programs, e.g., ImageJ or FIJI. As the 3D surface-rendered image is very large (712 MB), we have provided a version for web-based 3D visualization in Sketchfab, embedded in the GigaDB entry. For 3D printing, the STL model is also available in Thingiverse: https://www.thingiverse.com/thing:3233744.

## Abbreviations

2D: two dimensional; 3D: three dimensional; μCT: microcomputed tomography; CT: computed tomography: ROI: regions of interest; CoE: Centre of Excellence

## Author information

The dataset was acquired and compiled during the course of M.R.'s MSc thesis at University of Cape Town supervised by E.M.B. and W.L.T. M.R., E.M.B., and W.L.T. conceived and designed the project; S.L.R. and A.D.P. prepared the data for upload and contributed to the technical side of this study; M.R. wrote most of the final paper and coordinated with co-authors (E.M.B., W.L.T., S.L.R., and A.D.P) who performed critical revision of the manuscript.
