# Supplementary material for: A micro X-ray computed tomography dataset of fossil echinoderms in an ancient obrution bed: a robust method for taphonomic and palaeoecologic analyses
Source: Gigascience. 2018 Dec 7;8(3):giy156. doi: 10.1093/gigascience/giy156 (PMC6505446; doi:10.1093/gigascience/giy156)
Supplement: GIGA-D-18-00323.pdf [file giy156_giga-d-18-00323.pdf]

GigaScience

A micro X-ray computed tomography dataset of fossil echinoderms in an ancient obrution bed: a robust method for taphonomic and palaeoecologic analyses

--Manuscript Draft--

Manuscript Number:	GIGA-D-18-00323
Full Title:	A micro X-ray computed tomography dataset of fossil echinoderms in an ancient obrution bed: a robust method for taphonomic and palaeoecologic analyses
Article Type:	Data Note
Funding Information:	DST-NRF Centre Of Excellence In Palaeoscience — Mrs Wendy L. Taylor
Abstract:	Background<br><br>Taphonomic and palaeoecologic studies of obrution beds often employ conventional methods of investigation such as physical removal and extraction of fossils from their sedimentary rock matrix by mechanical preparation. This often-destructive method is not suitable for studying mouldic fossiliferous beds which, due to dissolution of the fossils in post-depositional processes and diagenesis, only contain impressions of the organisms in voids left in host sedimentary rocks.<br><br>Findings<br><br>Micro computed tomography (μCT) scan data of 25 fossiliferous rock samples revealed thousands of Paleozoic echinoderms and digitally 'stitching' together individually μCT scanned rock samples within 3D space allows for quantifiable taphonomic data on a fossil echinoderm-rich obrution deposit. A brief step-by-step guide is provided on creating, segmenting and ultimately combining sections of a richly fossiliferous bed to create a virtual model of the bed suited for the quantitative and qualitative taphonomic analysis of a marine benthic assemblage from the Devonian (Emsian) of South Africa.<br><br>Conclusions<br><br>The use of visualising the internal features of fossiliferous beds in 3D is an invaluable taphonomic tool for analysing delicate fossils, accounting for all specimens irrespective of their preservation stages and without damaging the. The technique is particularly useful for analysing fossiliferous deposits with highly mouldic fossils that prove to be difficult to study with traditional methods, because the method relies on the large density contrast between the natural cast and host rock.
Corresponding Author:	Mhairi Lesley Reid, Msc<br>University of Cape Town<br>Cape Town, Western Cape SOUTH AFRICA
Corresponding Author Secondary Information:	
Corresponding Author's Institution:	University of Cape Town
Corresponding Author's Secondary Institution:	
First Author:	Mhairi Lesley Reid, Msc
First Author Secondary Information:	
Order of Authors:	Mhairi Lesley Reid, Msc<br>Emese M. Bordy<br>Wendy L. Taylor<br>Stephan G. le Roux<br>Anton du Plessis

Powered by Editorial Manager® and ProduXion Manager® from Aries Systems Corporation

Order of Authors Secondary Information:	
Additional Information:	
Question	Response
Are you submitting this manuscript to a special series or article collection?	No
**Experimental design and statistics**<br><br>Full details of the experimental design and statistical methods used should be given in the Methods section, as detailed in our Minimum Standards Reporting Checklist. Information essential to interpreting the data presented should be made available in the figure legends.<br><br>Have you included all the information requested in your manuscript?	Yes
**Resources**<br><br>A description of all resources used, including antibodies, cell lines, animals and software tools, with enough information to allow them to be uniquely identified, should be included in the Methods section. Authors are strongly encouraged to cite Research Resource Identifiers (RRIDs) for antibodies, model organisms and tools, where possible.<br><br>Have you included the information requested as detailed in our Minimum Standards Reporting Checklist?	Yes
**Availability of data and materials**<br><br>All datasets and code on which the conclusions of the paper rely must be either included in your submission or deposited in publicly available repositories (where available and ethically appropriate), referencing such data using a unique identifier in the references and in the "Availability of Data and Materials" section of your manuscript.	Yes

Have you have met the above requirement as detailed in our Minimum Standards Reporting Checklist?	

Manuscript

Click here to download Manuscript REID_MS GIGAScience 230818.docx

A micro X-ray computed tomography dataset of fossil echinoderms in an ancient obrution bed: a robust method for taphonomic and palaeoecologic analyses

Mhairi Reid[1*], Emese M. Bordy[1], Wendy L. Taylor[1], Stephan G. le Roux[2], Anton du Plessis[2]

[1] Department of Geological Sciences, University of Cape Town, Cape Town, South Africa.

[2] CT Scanner Facility, Central Analytical Facilities, Stellenbosch University, Stellenbosch, South Africa.

*Corresponding Author: Mhairi Reid

E-mail : rdxmha001@myuct.ac.za

Emese M. Bordy [emese.bordy@uct.ac.za], Wendy L. Taylor [Wendy.L.Taylor@asu.edu], Stephan G. le Roux [lerouxsg@sun.ac.za], Anton du Plessis [anton2@sun.ac.za]

Abstract

Background: Taphonomic and palaeoecologic studies of obrution beds often employ conventional methods of investigation such as physical removal and extraction of fossils from their sedimentary rock matrix by mechanical preparation. This often-destructive method is not suitable for studying mouldic fossiliferous beds which, due to dissolution of the fossils in post-depositional processes and diagenesis, only contain impressions of the organisms in voids left in host sedimentary rocks. **Findings:** Micro computed tomography (µCT) scan data of 25 fossiliferous rock samples revealed thousands of Paleozoic echinoderms and digitally 'stitching' together individually µCT scanned rock samples within 3D space allows for quantifiable taphonomic data on a fossil echinoderm-rich obrution deposit. A brief step-by-step guide is provided on creating, segmenting and ultimately combining sections of a richly fossiliferous bed to create a virtual model of the bed suited for the quantitative and qualitative taphonomic analysis of a marine benthic assemblage from the Devonian (Emsian) of South Africa.
Conclusions: The use of visualising the internal features of fossiliferous beds in 3D is an invaluable taphonomic tool for analysing delicate fossils, accounting for all specimens irrespective of their preservation stages and without damaging the. The technique is particularly useful for analysing fossiliferous deposits with highly mouldic fossils that prove to be difficult to study with traditional methods, because the method relies on the large density contrast between the natural cast and host rock.

Key words: micro-CT, µCT, 3D imaging, virtual taphonomy, obrution deposit, echinoderms.

Data Description

Motivation and background

Microcomputed tomography (micro-CT or μCT) and three-dimensional (3D) visualisation techniques have become an increasingly popular tool used in many fields of palaeontological research [1-4] especially in anatomy and functional morphology of vertebrates [5-9], invertebrates [10-12] and even in micropalaeontology [13]. The advantage of this imaging technique lies in its power to construct high resolution, cross-sectional views of fossils causing damage [13,14]. Conventionally, palaeontologists use mechanical preparation techniques (e.g., air abrasive tools, pneumatic tools) which often damage delicate structures and seldom allow entire specimens to be completely exposed [1]. Moreover, many fossils are preserved as casts where the fossil itself was dissolved away by post-depositional processes leaving only a void in the host rock that was filled in with sediment. Anatomical details are captured only in the impressions of the external surfaces of the cast. Advancements of X-ray tomographic technology and data processing software (e.g., VG Studio Max and SPIERS) enable scientists to not only visualise two-dimensional (2D) dissections of scanned fossil material but also to reconstruct high-resolution 3D models of body fossils as well as casts from a variety of sedimentary rocks [2]. In recent years, X-ray based methods have been extensively applied in the analysis of invertebrate fossils of different sizes (from macro- to micro-morphology) [15-17]. The study of fossil echinoderms, a group of marine invertebrates that possesses delicate, multielement calcitic skeletons, has particularly benefited from the use of micro-CT techniques.

This dataset was created with the purpose of visualizing a complex fossiliferous obrution deposit, focusing on two types of bottom-dwelling fossil echinoderms, ophiuroids or brittle stars, with

many modern relatives that are common in oceans today, and an extinct group known as stylophorans. Obrution beds form during storms due to the sudden smothering of the seafloor-dwelling communities by rapidly deposited storm sediments (tempestites) [18,19]. By providing snapshots into the palaeoecology of marine organisms, obrution deposits often display not only exceptional fossil preservation, but also unusual or exceptional information that would otherwise be lost from the fossil record through destructive taphonomic processes (e.g., decay, disarticulation, fragmentation, transport, scavenging) [20]. Here, we introduce the potential of virtual reconstructions as a means of investigation into often complex fossiliferous obrution deposits, with focus on larger taphonomic assessments rather than investigating anatomical structures of individual fossils themselves. This µCT technique allowed the 3D visualisation of not only the degree of articulation for each individual specimen but also revealed different taxa present within the bed. Furthermore, the imaging of very small (1-3 mm) stylophorans that would have been missed with conventional study was only possible using this high-resolution scanning method. Taphonomic observations such as orientation (oral side up, down, or oblique) of the fossils within the bed, posture and arrangement of ophiuroid arms and their relative spatial arrangement to one another, could all be quantified *in situ*. In this Data Note, we also provide a brief step-by-step guide on creating, segmenting and ultimately combining sections of a fossiliferous layer containing abundant remains of delicate ophiuroids and stylophorans, in order to create a virtual view suited for the quantitative and qualitative taphonomic analysis. An accompanying publication presents the results of this analysis in Reid et al. [21].

Material and methods

Excavation of the fossil bed

The initial discovery of the Karbonaatjies bed occurred during a preliminary study in 2014, when collected samples revealed rare, well-preserved individuals of an undescribed taxon of ophiuroid and stylophorans [22]. The study area is located within the Cape Fold Belt and the exposed rocks lithostratigraphically belong to the Lower Devonian (Emsian ~400 Ma) Voorstehoek Formation, Bokkeveld Group in South Africa [23]. The obrution bed was excavated from a road-cutting on Karbonaatjies farm, which is situated ~145 km northeast of Cape Town (GPS: 33°24003.600S, 19°52042.700E). A section of the obrution bed, approximately 2 x 1 m wide with an average thickness of 4 cm, was systematically excavated using a flat brick chisel, geological hammer and pickaxe. The highly-weathered nature of the outcrop caused the obrution bed to break up into 55 pieces during removal and each piece was labelled from A to WW (Fig. 1). All pieces were carefully reassembled in the lab and photographed.

Conventional palaeontological analysis of this deposit posed problems due to the fragile state of the fossils caused by the deep chemical weathering of the originally calcitic fossils, leaving only voids with natural casts in the host rock, a silty, very fine-grained sandstone. For this reason, µCT scanning was used to taphonomically analyse the Karbonaatjies obrution bed, we discovered that the sampled portion of this layer contained over 1000 ophiuroid specimens of a new genus and species and hundreds of stylophorans, within a 2-3 cm thick lens of host rock.

Scanning, data processing and quality control

The µCT scanning was performed at the Stellenbosch University Central Analytical Facility with the aid of a walk-in microfocus X-ray CT scanner; the General Electric Phoenix V|Tome|X L24 model with additional NF180 option [24]. The CAF micro-CT instrument has a typical minimum

voxel size of between 1 and 100 μm and can be used for samples that are up to 300 mm long and 200 mm wide. Samples were placed within a plastic bottle supported by dense polystyrene foam for scanning. Typical considerations for scan setup and parameter choices were outlined by du Plessis et al. [24]. A small wax ball was stuck to the upper surface of each sample in order to indicate right way up orientation as well as the relative position to other samples within the bed. To ensure that the X-ray spot size did not exceed the selected scan resolution, optimal X-ray scan parameters were chosen while using live digital X-ray images (e.g., for ideal X-ray penetration, we monitored the high transmitted brightness values). X-ray settings ranged for voltage from 160 kV to 240 kV (for larger samples) and for current from 200 μA to 220 μA depending on sample size respectively. Detector shift was activated and background calibration was performed before each scan in order to minimize ring artefacts and achieve good high image contrast. To reduce potential beam hardening artefacts, a 0.1 mm copper beam filter was used in all the scans. The samples in this study are relatively dense, rigid sandstone fragments with an average length of 200 mm. To obtain sharp images of larger samples, the voltage had to be raised up to 240 kV on the high-power tube allowing for more beam filtration (less beam hardening) and limiting the generation of other artefacts [25]. Scan time averaged from approximately 40-60 minutes per scan depending on the size of the sample. Longer samples were scanned in sections (allowing for higher magnification). Using an exposure time of 500 ms per image, images were acquired in steps during a full 360° rotation. At each step position, the first image was discarded and the next two images averaged to obtain lower noise and sharper images. The acquired projection images (between 1400 and 2600 images per scan) were reconstructed using system-supplied Datos reconstruction software, where the choice of numbers of projections depends on sample size and magnification and was selected according to the guidelines in du Plessis et al. [24].

Digital analysis

The tomographic reconstruction dataset obtained from scanning was visualised and analysed using the VGStudioMax 3.1 software package (website: http://volumegraphics.com) to create a 3D view of individual fossils within each sample. This software was also used to produce images (e.g., screenshots) and animations.

Virtual preparation and dissection of the specimens involved a series of modified steps outlined by Abel [26] and Matthews [27]: (1) Density contrast enhancement: generation of a larger contrast between the grey scale values that represent the rock and surrounding air, by optimizing the grey value range on the histogram; (2) Register object: alignment of the sample to a specific coordinate system so that the top-down 2D viewer of the slices scrolls through the sample parallel to the bedding plane; (3) Surface determination: defining the material boundary of interest. This is generally the quickest and easiest way to separate a region of interest (ROI), however this was not possible because the fossils (preserved mostly as void space) have the same density or grey scale value as the permeating cracks in the samples and the surrounding air. It is for this reason that the region growing tool was predominantly used; (4) Region growing: generating a selection using a region growing algorithm (Fig. 2A). This is one of the simpler image segmentation methods used for 3D data, which essentially establishes the ROI (i.e., subparts of the volume data). The region growing tool allows the selection of a 'seed' point (in this case the 'black' voxel of a fossil or the voids within the rock sample), the algorithm will expand the selection to all voxels connected to that seed point based on a defined tolerance of voxel grey values relative to the selected seed point. The threshold (selection of voxels with grey

values within the selected grey value interval) changed from sample to sample but was generally around ±5000 in this study. Region growing was the most time-consuming step as each individual fossil had to be segmented out in order to make the 3D volume rendering. Generally, when a fossil is scanned it is the minerals that make up the rock and the fossil itself that are compositionally differentiated so there is enough contrast to allow anatomical structures to be digitally visualised in 3D. This highlights one of the difficulties with CT scanning fossiliferous rock samples. If the compositional difference between the fossils and the sedimentary rock matrix is negligible, little or no information will be captured in the scans. However, the fossils of this study are predominantly casts (i.e., large voids in the host rock) with no internal mineralogical information, and this made segmentation much easier in that the contrast between the fossil (air filled void) and surrounding rock are very high. Finally, the last step involved (5) Volume rendering: generation of a 3D volume from the segmented 2D ROI's using a specific rendering algorithm in this case, the isosurface render. Isosurfaces are mathematically defined surfaces calculated from a volume along points of interest [3]. The dataset is treated as a volume comprising voxels (3D pixels that contain measurements of colour) instead of 2D pixels. Once the volume is created, the appearance of the volume objects can be manipulated (e.g., colour, transparency) to visualise the fossils in 3D (Fig. 2B). Lastly, once the fossils were segmented out, rendered in 3D and false-coloured accordingly, the fossiliferous rock samples were virtually 'stitched' together to recreate a section of the obrution bed (Fig. 2C). This was done by using the volume import tool, each sample had to be manually aligned and placed in 3D space.

Data quality and limitations

In spite of the general preventative measures (e.g., using a copper filter and scanning perpendicular to the long axis of the sample), a number of the larger and longer samples scanned had artefacts which obscured details in the CT images. This makes interpretation and analysis very difficult, and sometimes even impossible (Fig. 3A, B). The major type of artefact identified is beam hardening which is a problem that arises when a high energy polychromatic X-ray source is used to penetrate a dense sample. The strong absorption of the beam in a large sample causes low-energy photons to be absorbed more strongly that high-energy photons, resulting in unequal absorptions giving rise to this type of artefact. This problem often occurs in palaeontological samples because of the high density and large size of the fossil specimens and their matrix, resulting in low transmission and high noise [3]. In our study, it made identification and separation of the echinoderms arduous and even impossible in some cases, as more details towards the center of the samples were lost. To rectify this, many samples were cut into smaller sections and most of the non-fossiliferous sedimentary matrix was removed (Fig. 3C). These samples were subsequently re-scanned in sections to obtain higher magnification, especially for longer samples. Longer samples are scanned using a vertical multiscan procedure whereby different parts are scanned automatically with some overlap and the reconstruction software performs automatic stitching of multiple scans according to the accurate vertical translation distances used (no manual interface is necessary). However, due to the high power required, the X-ray source often became unstable causing errors and failed scans. For this reason, some individual parts of larger samples were scanned separately and manually stitched. The advantage of this is that if there is a failure only one part needs to be rescanned, and less overlap is required for manual stitching, reducing the number of scans required for long or large objects. Finally,

while all 55 samples were CT scanner, time and funding constraints allowed for only half of the bed (25 samples) to be rendered in 3D.

Potential uses

The dataset presented can be used as an example of how taphonomic and palaeoecological investigations can be conducted using µCT scanned rock samples within 3D space. Colourising the different taxa present in the virtually reconstructed fossiliferous bed played an important role in the taphonomic assemets. For example, ophiuroids were colour-coded according to their different orientations, light pink for oral (mouth) side up and dark pink for oral side down their presumed life position (Fig. 4A). Quantifying the percentage of ophiuroids in life position is a possible indication of the extent to which the ophiuroids may have been transported by storm-induced current and are subsequently reoriented before burial. Using the 3D reconstruction of the bed, most ophiuroids and stylophorans could be assigned to different taphonomic groups or decay stages based on their level of preservation. For example, ophiuroids that have fully articulated arms or stylophorans with a complete theca and aulacophore can be assigned to Group 1, which is intact or complete preservation. Other taphonomic categories were used for specimens in more fragmentary stages of preservation. Palaeoecological and taxonomic measurements such as specimen counts, ophiuroid disc or body diameter measured from the base of the arm to the opposite interradius, ophiuroid arm length measured in relation to the disc diameter, stylophoran theca or body width and length, were all measured directly onto the 2D tomographic images using the digital calliper tool in VGStudioMax.

One of the unique perspectives of virtually viewing the obrution deposit in 3D is that it allows the examination of multiple levels of fossils preserved *in situ* within the bed (Fig. 4B). This is of particular interest because it provides insights into the transport medium that ultimately smothered the echinoderms. In this case, the dense assemblage of ophiuroids is arranged into vaguely laminated horizons with associated fossil shell debris. This indicates important information about the initial pre-burial storm conditions and the palaeoecologic fidelity of the resulting deposit. By setting the surrounding matrix to transparent in the program, other features such as the different arrangements of ophiuroid arms and the flexure of the sylophorans can be seen within the 3D space. One of the most striking features of the obrution bed was identified by using this technique. We observed that many of the ophiuroids had one or more arms extended upward into the overlying sediment. This has been identified as an escape posture and is comparable to modern examples of ophiuroids escaping from an influx of sediment [28]. Evidence of this behaviour in ancient ophiuroids is often difficult to interpret and is rarely observable in preserved ancient ophiuroids because traditional methods do not allow for such a comprehensive 3D view of the fossils [29,30] .

The use of micro-CT scanning in palaeontological research has grown by leaps and bounds over the past 10 years. In this study, it allowed the recognition of small, cryptic fossil taxa that would have been otherwise missed as well as the observation of key palaeontological features that are critical to the interpretation of the deposit.

Availability of supporting data

The presented microCT data are available as image stacks for each sample scanned and the final 3D render of the surface and all fossils is available as a .vgl file which can be viewed on the free downloadable mvVGL program at https://www.volumegraphics.com/en/products/myvgl.html.

Abbreviations

3D: three dimensional; 2D: two dimensional; μCT: micro-computed tomography; CT: computed tomography: ROI: regions of interest.

Competing interests

The authors declare that they have no competing interests.

Authors' information

The dataset was compiled during the course of MR's Masters thesis supervised by EB and WT. MR, EB, WT conceived and designed the project. SLR and ADP prepared the data for upload and contributed to the technical side of this study.

Acknowledgements

We are grateful to Sandra Engels from Volume Graphics Gmbh for providing us with VGStudio Max at UCT. We gratefully acknowledge these financial contributions. This project was supported by grants to WT and EB as well as postgraduate funding to MR from the DST-NRF Centre of Excellence in Palaeosciences (CoE in Palaeosciences). Opinions expressed and conclusions arrived at are those of the authors and are not necessarily to be attributed to COE PAL

References

1. Sutton MD. Tomographic techniques for the study of exceptionally preserved fossils: Proceedings of the Royal Society. Series B. 2008; 275: 1587–1593. https://doi.org/10.1098/rspb.2008.0263
2. Cunningham JA, Rahman IA, Lautenschlager S, Rayfield EJ, Donoghue PCJ. A virtual world of paleontology: Trends in Ecology and Evolution. 2014; 29: 347–357. https://doi.org/10.1016/j.tree.2014.04.004
3. Sutton M, Rahman I, Garwood R. Techniques for virtual palaeontology. John Wiley & Sons; 2014 Feb 5.
4. Sutton MD, Rahman IA, Garwood RJ. Virtual paleontology—An overview: Paleontological Society Special Papers. 2017; 22:1–20.
5. Burrow CJ, Jones AS, Young GC. X-ray microtomography of 410 million-year-old optic capsules from placoderm fishes. Micron. 2005; 36(6): 551-557. https://doi.org/10.1016/j.micron.2005.05.005
6. Claessens LP, O'Connor PM, Unwin DM. Respiratory evolution facilitated the origin of pterosaur flight and aerial gigantism. PloS One: 2009; 4(2): e4497. https://doi.org/10.1371/journal.pone.0004497
7. Rossi M, Casali F, Romani D, Bondioli L, Macchiarelli R, Rook L. MicroCT Scan in paleobiology: application to the study of dental tissues. Nuclear Instruments and Methods in Physics Research Section B: Beam Interactions with Materials and Atoms. 2004; 747-750. https://doi.org/10.1016/S0168-583X(03)01697-5

8. Coates MI, Gess, RW, Finarelli JA, Criswell KE, Tietjen K. A symmoriiform chondrichthyan braincase and the origin of chimaeroid fishes. Nature. 2017; 541 (7636): 208-211. https://doi.org/10.1038/nature20806

9. Lautenschlager S. Fossils explained 69: From bone to pixel—fossil restoration and reconstruction with digital techniques. Geology today. 2017; 33(4): 155-159. https://doi.org/10.1111/gto.12194

10. Stock SR, Veis A. Preliminary microfocus X-ray computed tomography survey of echinoid fossil microstructure. Geological Society Special Publication. 2003; 215: 225-235. https://doi.org/10.1144/GSL.SP.2003.215.01.21

11. Meyer M, Elliott D, Wood AD, Polys NF, Colbert M, Maisano JA, Vickers-Rich P, Hall M, Hoffman KH, Schneider G, Xiao S. Three-dimensional microCT analysis of the Ediacara fossil *Pteridinium simplex* sheds new light on its ecology and phylogenetic affinity. Precambrian Research. 2014; 249: 79-87. https://doi.org/10.1016/j.precamres.2014.04.013

12. Lee, Sangmin, Shi GR, Park, Tae-Yoon S, Oh, Jae-Ryong, Mii, Horng-Sheng, Lee, Mirinae. Virtual palaeontology: the effects of mineral composition and texture of fossil shell and hosting rock on the quality of X-ray microtomography (XMT) outcomes using Palaeozoic brachiopods. Palaeontologia Electronica. 2017; 20.2.3T: 1-25.

13. Görög A, Szinger B, Tóth E, Viszkok J. Methodology of the micro-computer tomography on foraminifera. Palaeontologia Electronica. 2012; 15(1): 15.

14. Garwood RJ, Rahman IA, Sutton MD. From clergymen to computers – the advent of virtual palaeontology. Geology Today. 2010; 26: 96–100. https://doi.org/10.1111/j.1365-2451.2010.00753.x

15. Hamada T, Tateno S, Suzuki N. Three dimensional reconstruction of fossils with X-ray and computer graphics. Scientific Papers of the College of Arts and Sciences Univ. Tokyo. 1991; 41: 107–118.

16. Dominguez P, Jacobson AG, Jefferies RPS. Paired gill slits in a fossil with a calcite skeleton. Nature. 2002; 417: 841–844. https://doi.org/10.1038/nature00805

17. Garwood RJ, Dunlop JA. Morphology and systematics of Anthracomartidae (Arachnida: Trigonotarbida). Palaeontology. 2011; 54: 145–161. https://doi.org/10.1111/j.1475-4983.2010.01000.x

18. Donovan SK. The Processes of Fossilization. London; Belhaven Press. 1991; 303.

19. Brett CE, Moffat HA, Taylor WL. Echinoderm taphonomy, taphofacies, and lagerstätten. In: Waters, J.A., Maples, C.G. (Eds.), Geobiology of Echinoderms. The Paleontological Society Papers. 1997; 3: 147–190.

20. Seilacher A, Reif WE, Westphal F. Sedimentological, ecological and temporal patterns of fossil-Lagerstätten. Philosophical Transactions of the Royal Society of London. 1985; 311: 5-23. https://doi.org/10.1098/rstb.1985.0134

21. Reid M, Taylor WL, Bordy EM, Brett CE, Hunter AW. Taphonomy and paleoecology of an ophiuroid-stylophoran obrution deposit from the Lower Devonian Bokkeveld Group, South Africa. PALAIOS. In review.

22. Reid M, Bordy EM, Taylor W. Taphonomy and sedimentology of an echinoderm obrution bed in the Lower Devonian Voorstehoek Formation (Bokkeveld Group, Cape Supergroup) of South Africa. Journal of African Earth Sciences. 2015; 110: 135-149. https://doi.org/10.1016/j.jafrearsci.2015.04.009

23. Rust IC. The Evolution of the Paleozoic Cape Basin, Southern Margin of Africa. In: A.E.M. Nairn and F.G. Stehli (eds.), The Ocean Basins and Margins. 1: The South Atlantic, Plenum Publishing Corp, New York, U.S.A. 1973; 247-276. https://doi.org/10.1007/978-1-4684-3030-1_6

24. du Plessis, A., le Roux, S. G., and Guelpa, A. The CT Scanner Facility at Stellenbosch University: an open access X-ray computed tomography laboratory. Nuclear Instruments and Methods in Physics Research Section B: Beam Interactions with Materials and Atoms. 2016; 384: 42-49.

25. Donovan SK. The Processes of Fossilization. London; Belhaven Press. 1991; 303.

26. Abel RL, Laurini CR, Richter M. A palaeobiologist's guide to 'virtual' micro-CT preparation. Palaeontologia Electronica. 2012; 15: 1-17.

27. Matthews T, du Plessis A. Using X-ray computed tomography analysis tools to compare the skeletal element morphology of fossil and modern frog (*Anura)* species. Palaeontologia Electronica. 2016; 19: 1-46.

28. Ishida Y, Fujita T. Escape behavior of epibenthic ophiuroids buried in the sediment: observations of extant and fossil Ophiura sarsii sarsii. In: Proceedings of the 10th International Echinoderm Conference. Dunedin 2000; 285-292.

29. Brett CE. Sedimentology, facies and depositional environments of the Rochester shale (Silurian, Wenlockian) in Western New York and Ontario. Journal of Sedimentary Petrology. 1983; 53(3): 947-972.

30. Jagt JWM. Late Cretaceous-Early Palaeogene echinoderms and the K/T boundary in the southeast Netherlands and northeast Belgium - Part 3: Ophiuroids. With a chapter on:

Early Maastrichtian ophiuroids from Rügen (northeast Germany) and Møn (Denmark) by Manfred Kutscher and John W.M. Jagt. Scripta Geologica. 2000; 121: 1-179.

Figure captions

Figure 1: Excavated Karbonaatjies obrution bed. Each individual rock fragment was given a reference letter in the field and subsequently reassembled in the lab.

Figure 2: A) Sample II, a long sample (26 cm by 12 cm) illustrating beam hardening artefacts causing the centre of the sample to appear to have darker voxels in the centre while the edges appear much brighter, even though the sample is homogeneous. B) Side view of Sample II showing how beam hardening artefact causes the pyrite minerals to give a ‘star-burst’ appearance. C) Sample II after being cut and rescanned following the above-mentioned procedure.

Figure 3: A) Segmentation of ophiuroid specimens using the region growing tool in VGStudioMax. B) The resulting virtually reconstructed ophiuroids in 3D, rendered with colour and surrounding matrix set to transparent. C) Reconstruction of a portion of the fossil bed in 3D (shaded area in Figure 1). Black arrows point towards wax balls stuck to the upper surface of the samples to indicate right way up and relative positions.

Figure 4: A) Virtual reconstruction of samples SS, TT, TT2, TT3 and UU2 rendered with lights, colour and surrounding matrix set to 90% transparent. Approximately 80 articulated ophiuroids (light pink = ophiuroids oral side down; dark pink = ophiuroids oral side up); 13 paranacystids

(green); one *Placocystella* (red) aulacophore fragment; numerous crinoid ossicles (blue); fragmented ophiuroid arms (pale orange) and large shell fragments (yellow) are all shown in 3D. B) Side view shows three vaguely defined ophiuroid ‘horizons’ as well as ophiuroid arms extended upward within the obrution bed.

Figure 1

Click here to download Figure Figure 1.tif

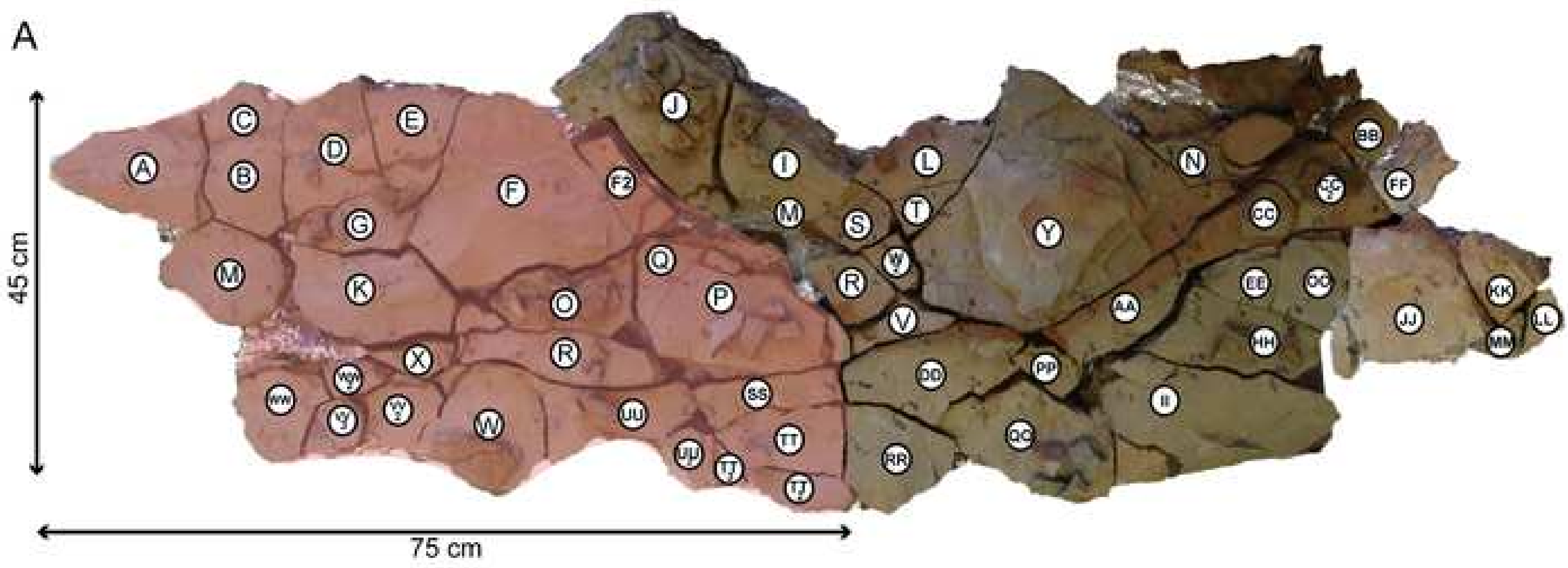

Figure 2

Click here to download Figure Figure 2.tif

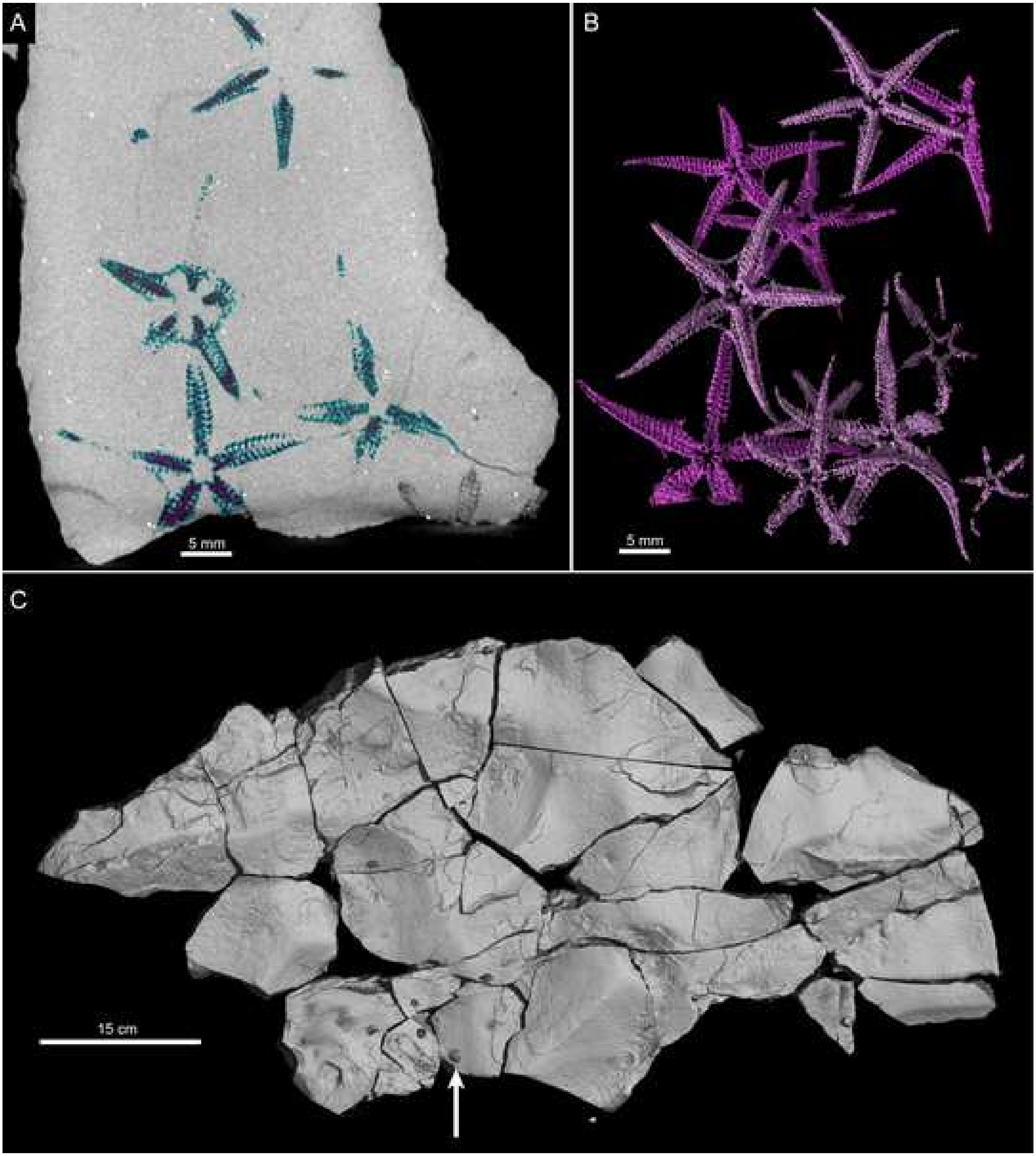

Figure 3 Click here to download Figure Figure 3.tif

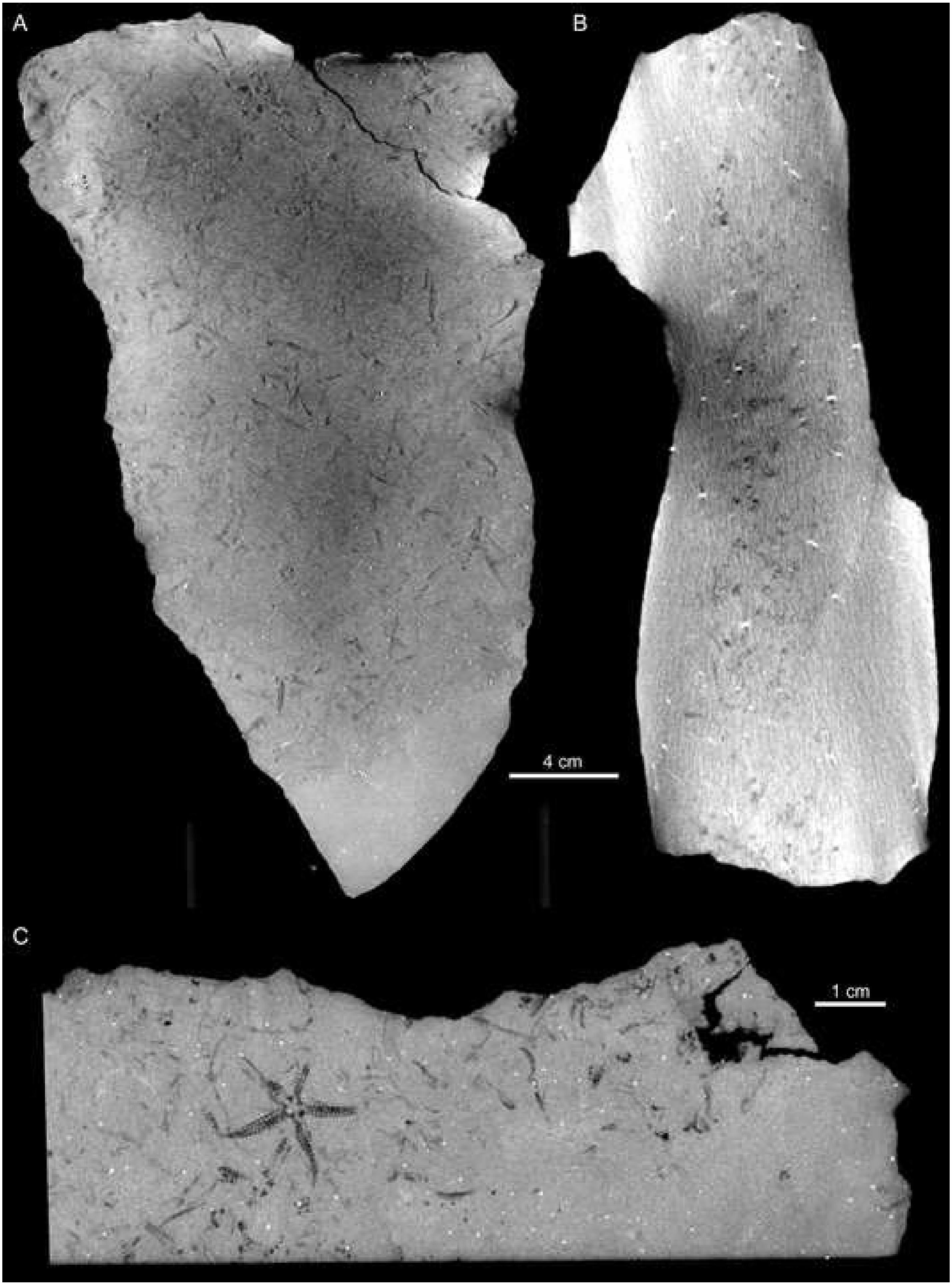

Figure 4 Click here to download Figure Figure 4.tif

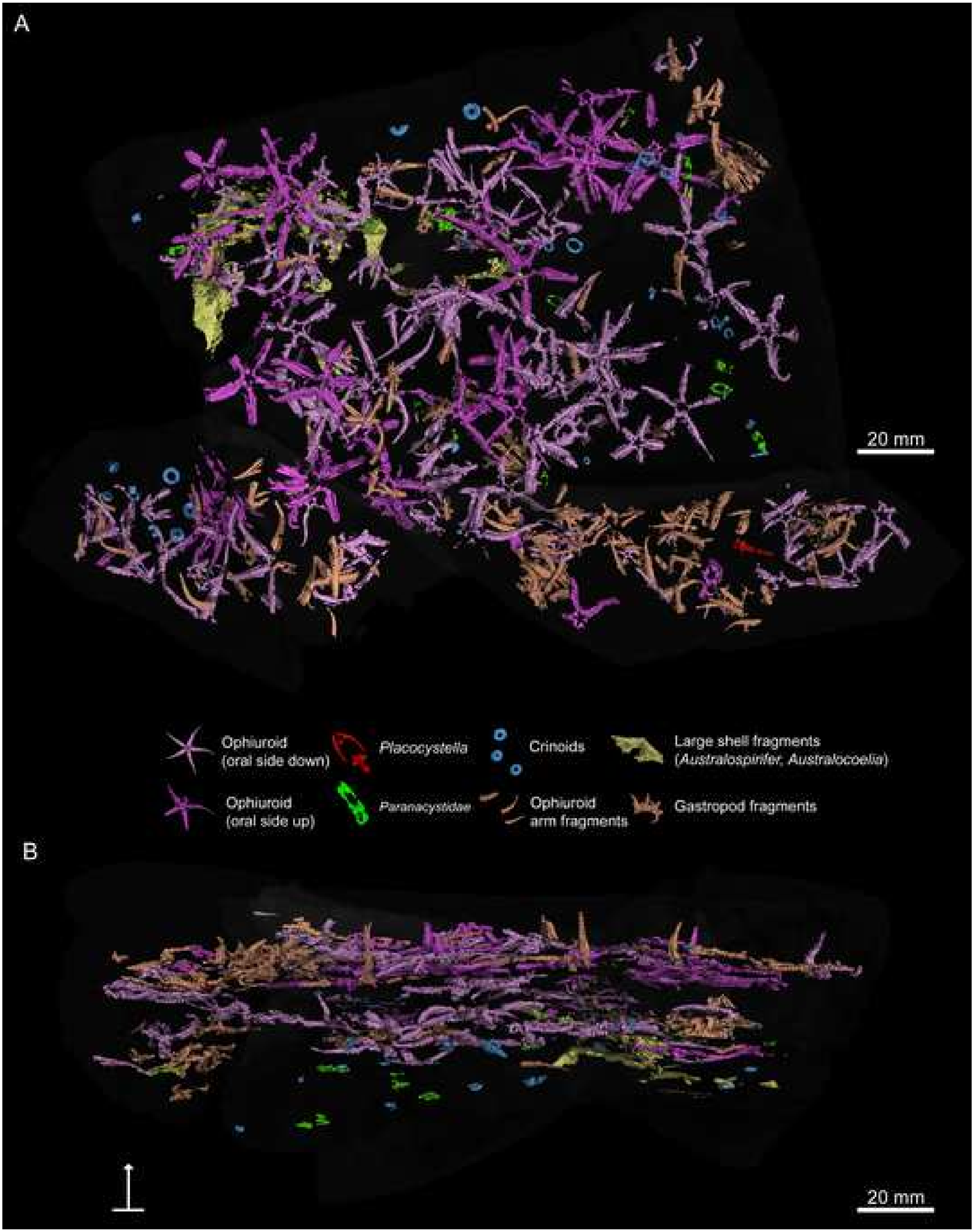

DEPARTMENT OF GEOLOGICAL SCIENCES • e-mail: rdxmha001@myuct.ac.za
13 University Avenue, Upper Campus - University of Cape Town, Rondebosch, 7701, South Africa

August 28, 2018

Dear Editor,

We wish to submit an original Data Note article “A micro X-ray computed tomography dataset of fossil echinoderms in an ancient obrution bed: a robust method for taphonomic and palaeoecologic analyses” for consideration by GigaScience.

We confirm that this work is original and has not been published elsewhere, nor is it currently under consideration for publication elsewhere.

In this Data Note, we provide a dataset that was created with the purpose of visualising a complex fossiliferous obrution deposit, focusing on two types of bottom-dwelling fossil echinoderms, ophiuroids (brittle stars) and an extinct group known as stylophorans. In this manuscript we provide a brief step-by-step guide on creating, segmenting and ultimately combining sections of a fossiliferous bed in order to create a virtual view suited for the quantitative and qualitative taphonomic analysis of a fossil bed. By utilising micro X-ray computed tomography (µCT) techniques, we take a novel approach of digitally ‘stitching’ together µCT scanned rock samples within 3D space in order to acquire quantifiable taphonomic data on a fossil echinoderm-rich obrution deposit.

We believe that this manuscript is appropriate for publication by GigaScience because our manuscript provides an example (the dataset) of how taphonomic and palaeoecological investigations can be conducted using µCT scanned rock samples within 3D space. This technique is particularly useful for analysing fossiliferous deposits with highly mouldic fossils that prove to be difficult to study with traditional methods. An accompanying publication will present the results of this analysis in Reid M, Taylor WL, Bordy EM, Brett CE, Hunter AW. Taphonomy and paleoecology of an ophiuroid-stylophoran obrution deposit from the Lower Devonian Bokkeveld Group, South Africa. PALAIOS. In review.

The dataset will be presented as microCT scan data (image stacks for each sample) as well as the 3D render of all fossils (ophiuroids etc.) within the fossil bed.

We have no conflicts of interest to disclose and confirm all authors have approved the manuscript.

Please address all correspondence concerning this manuscript to me at rdxmha001@myuct.ac.za

Thank you for your consideration of this manuscript.

Yours truly,

Mhairi Reid
PhD student – Geology
http://www.geology.uct.ac.za/mhairi/reid/research
