# Supplementary material for: A micro X-ray computed tomography dataset of fossil echinoderms in an ancient obrution bed: a robust method for taphonomic and palaeoecologic analyses
Source: Gigascience. 2018 Dec 7;8(3):giy156. doi: 10.1093/gigascience/giy156 (PMC6505446; doi:10.1093/gigascience/giy156)
Supplement: GIGA-D-18-00323_R1.pdf [file giy156_giga-d-18-00323_r1.pdf]

GigaScience

A micro X-ray computed tomography dataset of fossil echinoderms in an ancient obrution bed: a robust method for taphonomic and palaeoecologic analyses

--Manuscript Draft--

Manuscript Number:	GIGA-D-18-00323R1
Full Title:	A micro X-ray computed tomography dataset of fossil echinoderms in an ancient obrution bed: a robust method for taphonomic and palaeoecologic analyses
Article Type:	Data Note
Funding Information:	DST-NRF Centre Of Excellence In Palaeoscience — Mrs Wendy L. Taylor
Abstract:	Background<br><br>Taphonomic and palaeoecologic studies of obrution beds often employ conventional methods of investigation such as physical removal and extraction of fossils from their host rock (matrix) by mechanical preparation. This often-destructive method is not suitable for studying mould fossils, which are voids left in host rocks due to dissolution of the original organism in post-depositional processes.<br><br>Findings<br><br>Micro computed tomography (µCT) scan data of 25 fossiliferous rock samples revealed thousands of Palaeozoic echinoderms, and digitally 'stitching' together individually µCT scanned rock samples within 3D space allows for quantifiable taphonomic data on a fossil echinoderm-rich obrution deposit from the Devonian (Emsian) of South Africa. A brief step-by-step guide is provided on creating, segmenting and ultimately combining sections of richly fossiliferous beds to create virtual models suited for the quantitative and qualitative taphonomic analyses of fossil invertebrate assemblages.<br><br>Conclusions<br><br>Visualizing the internal features of fossiliferous beds in 3D is an invaluable taphonomic tool for analysing delicate fossils, accounting for all specimens irrespective of their preservation stages and with minimal damage. This technique is particularly useful for analysing fossiliferous deposits with mould fossils that prove to be difficult to study with traditional methods, because the method relies on the large density contrast between the mould and host rock.
Corresponding Author:	Mhairi Lesley Reid, Msc<br>University of Cape Town<br>Cape Town, Western Cape SOUTH AFRICA
Corresponding Author Secondary Information:	
Corresponding Author's Institution:	University of Cape Town
Corresponding Author's Secondary Institution:	
First Author:	Mhairi Lesley Reid, Msc
First Author Secondary Information:	
Order of Authors:	Mhairi Lesley Reid, Msc
	Emese M. Bordy
	Wendy L. Taylor
	Stephan G. le Roux
	Anton du Plessis
Order of Authors Secondary Information:	

Powered by Editorial Manager® and ProduXion Manager® from Aries Systems Corporation

**Response to Reviewers:**	Responses to Reviewers<br><br>Reviewer #1: This is a novel and interesting study that utilises microcomputed tomography (micro-CT) to image complex echinoderm fossil specimens. The manuscript is well written, and the authors demonstrate quite beautifully how it is possible to use non-destructive 3D imaging techniques to segment starfish fossils from the host rock. Segmentations used in this study were accomplished using the commercial package VG Studio Max. The authors additionally provide an extremely useful step-by-step guide on how to generate these segmentations. From the figures, the 3D reconstructions look of sufficient quality for GigaDB to be able to generate surface reconstructions that can be interactively explored on the GigaDB website. Consequently, I suggest that GigaScience / GigaDB generate 3D models for this dataset to allow GigaDB users to interactively explore the echinoderm 3D reconstructions in the context of a web browser.<br><br>Response: We have now a STL file of the entire segmented fossil bed for this purpose. This process required creating meshes of all volumes and combining the meshes into one, using the "normal with simplification" meshing option.<br><br>Reviewer #1: Whereas the manuscript is well-written, the image data were submitted in proprietary formats that have some serious drawbacks in terms of accessibility and re-usability. The authors have submitted the volumetric image data in a proprietary image format that does not open with standard image viewers such as ImageJ or FIJI. I strongly encourage that the authors make the data available in a format that is more widely used (e.g. TIFF-stack) so that they are accessible (open, free, and universally implementable), and reusable (data are released with a clear and accessible data usage license). In this respect, I think it would be particularly useful if the authors make the image stacks available in a format that can be easily read by ImageJ or FIJI. Likewise, I suggest that the authors make the surface reconstruction file available in a more widely used format, such as OBJ or STL, rather than the proprietary VGL format that they have used. This will greatly encourage re-use of these data. Consequently, it is my recommendation that, in addition to VG Studio Max proprietary formats, the authors submit the supporting image data in more widely accessible formats.<br><br>Response: Thank you for the recommendation and we agree in making the image data more accessible. We submitted in VG Studio format which is possible to be accessed using their free viewer program, so is accessible to anyone who wishes, in windows or mac versions. The reason for this submission format is that the segmentation was performed in this software and the viewing of the segmented coloured data in ROIs is possible in this format. We also submitted Tiff stacks in the first instance so this should be accessible in imageJ, maybe the reviewer missed those? Finally, we now have made a STL of the entire segmented fossil bed. We hope it is now accessible and open to all!<br><br>Reviewer #2: Micro computed tomography (micro-CT) is by no means a novel technique in the field of Palaeontology. Well, you may be the first ones who apply this technique to the research of that Devonian assemblage from South Africa, but in the meantime, you basically follow the others' workflow published in the last years. I cannot see any innovation or improvements in the "Methods" section. The dataset can be useful for the research of the assemblage, though. Therefore, I feel a journal in the field of Palaeontology is more suitable for this manuscript.<br><br>Response: While we agree that this not a novel technique, we feel that the application of the technique is different from the conventional approach. This is a Data Note paper demonstrating the largest combination of aligned microCT data to date to our knowledge, virtually recreating a fossil bed with large numbers of segmented features. We feel strongly it adds value, showing what is now possible with microCT and are very happy to have GigaScience reproduce our data so that everyone may have access to the digitized fossil bed. We currently have another paper in review in a paleontological journal on the taphonomy and paleoecology of the obrution bed illustrating the results of the microCT data obtained in detail.<br><br>Reviewer #3: I had reviewed a previous version of this manuscript and this is a much revised edition. A well put together MS!

	Response: thank you, we appreciate the positive comment!
**Additional Information:**	
**Question**	**Response**
Are you submitting this manuscript to a special series or article collection?	No
**Experimental design and statistics**<br><br>Full details of the experimental design and statistical methods used should be given in the Methods section, as detailed in our Minimum Standards Reporting Checklist. Information essential to interpreting the data presented should be made available in the figure legends.<br><br>Have you included all the information requested in your manuscript?	Yes
**Resources**<br><br>A description of all resources used, including antibodies, cell lines, animals and software tools, with enough information to allow them to be uniquely identified, should be included in the Methods section. Authors are strongly encouraged to cite Research Resource Identifiers (RRIDs) for antibodies, model organisms and tools, where possible.<br><br>Have you included the information requested as detailed in our Minimum Standards Reporting Checklist?	Yes
**Availability of data and materials**<br><br>All datasets and code on which the conclusions of the paper rely must be either included in your submission or deposited in publicly available repositories (where available and ethically appropriate), referencing such data using a unique identifier in the references and in the "Availability of Data and Materials"	Yes

section of your manuscript.<br><br>Have you have met the above requirement as detailed in our Minimum Standards Reporting Checklist?	

Manuscript
Click here to access/download;Manuscript;REID_MS GIGAScience revised 161018_SCE(1).docx
Click here to view linked References

A micro X-ray computed tomography dataset of fossil echinoderms in an ancient obrution bed: a robust method for taphonomic and palaeoecologic analyses

Mhairi Reid[1*], Emese M. Bordy[1], Wendy L. Taylor[1], Stephan G. le Roux[2], Anton du Plessis[2]

[1] Department of Geological Sciences, University of Cape Town, Cape Town, South Africa.

[2] CT Scanner Facility, Central Analytical Facilities, Stellenbosch University, Stellenbosch, South Africa.

*Corresponding Author: Mhairi Reid

E-mail : rdxmha001@myuct.ac.za , ORCID: 0000-0001-5169-2180

Emese M. Bordy [emese.bordy@uct.ac.za], ORCID: 0000-0003-4699-0823; Wendy L. Taylor [wendy.taylor@uct.ac.za], ORCID: /0000-0002-9899-5864; Stephan G. le Roux [lerouxsg@sun.ac.za], ORCID : 0000-0002-5617-8137; Anton du Plessis [anton2@sun.ac.za], ORCID : 0000-0002-4370-8661

Abstract

Background: Taphonomic and palaeoecologic studies of obrution beds often employ conventional methods of investigation such as physical removal and extraction of fossils from their host rock (matrix) by mechanical preparation. This often-destructive method is not suitable for studying mould fossils, which are voids left in host rocks due to dissolution of the original organism in post-depositional processes. **Findings:** Micro computed tomography (µCT) scan data of 24 fossiliferous rock samples revealed thousands of Palaeozoic echinoderms, and digitally 'stitching' together individually µCT scanned rock samples within 3D space allows for quantifiable taphonomic data on a fossil echinoderm-rich obrution deposit from the Devonian (Emsian) of South Africa. A brief step-by-step guide is provided on creating, segmenting and ultimately combining sections of richly fossiliferous beds to create virtual models suited for the quantitative and qualitative taphonomic analyses of fossil invertebrate assemblages. **Conclusions:** Visualizing the internal features of fossiliferous beds in 3D is an invaluable taphonomic tool for analysing delicate fossils, accounting for all specimens irrespective of their preservation stages and with minimal damage. This technique is particularly useful for analysing fossiliferous deposits with mould fossils that prove to be difficult to study with traditional methods, because the method relies on the large density contrast between the mould and host rock.

Key words: micro-CT, µCT, 3D imaging, virtual taphonomy, obrution deposit, echinoderms.

Data Description

Motivation and background

Microcomputed tomography (micro-CT or μCT) and three-dimensional (3D) visualisation techniques have become an increasingly popular tool used in many fields of palaeontological research [1-4] especially in anatomy and functional morphology of vertebrates [5-9], invertebrates [10-12] and even in micropalaeontology [13]. The advantage of this imaging technique lies in its power to construct high resolution, cross-sectional views of fossils without causing damage during extraction from the rock matrix [13, 14]. Conventionally, palaeontologists use mechanical preparation techniques (e.g., air abrasive tools, pneumatic tools) which often damage delicate fossil structures and seldom allow entire specimens to be completely exposed [1]. Moreover, many fossils are preserved as moulds where the fossil itself was dissolved away by post-depositional processes leaving only a void in the host rock. Anatomical details are captured only in the impressions of the external surfaces of the original fossil. Advancements of X-ray tomographic technology and data processing software (e.g., VG Studio Max and SPIERS) enable scientists to not only visualise two-dimensional (2D) dissections of scanned fossil material but also to reconstruct high-resolution 3D models of body fossils as well as mould fossils from a variety of host rocks [2]. In recent years, X-ray based methods have been extensively applied in the morphological analysis of both macro- to micro-size invertebrate fossils [15-17]. The study of fossil echinoderms, a group of marine invertebrates characterized by delicate, multielement calcareous skeletons, has particularly benefited from the use of micro-CT techniques.

This dataset was created with the purpose of visualizing a complex fossiliferous obrution deposit, and particularly focusing on two types of seafloor-dwelling fossil echinoderms found in this rock layer: a) ophiuroids or brittle stars, with many modern relatives that are common in oceans today, and 2) an extinct group known as stylophorans. Obrution beds form during storms due to the sudden smothering of the seafloor-dwelling communities (marine benthos) by rapidly deposited storm sediments (tempestites) [18, 19]. By providing snapshots into the palaeoecology of marine organisms, obrution deposits often display not only exceptional fossil preservation, but also rare behavioral information that would otherwise be lost from the fossil record through destructive taphonomic (fossilization) processes (e.g., decay, disarticulation, fragmentation, transport, scavenging) [20]. Here, we introduce the utility of virtual reconstructions as a means of investigating often complex fossiliferous obrution deposits, with focus on taphonomic assessments of the fossil community rather than investigating anatomical structures of individual fossils themselves. This µCT technique allowed the 3D visualisation of not only the degree of articulation for each individual specimen but also revealed different taxa and relationship to other taxon present within the bed. Furthermore, the imaging of very small (1-3 mm) stylophorans that would have been missed with conventional study was only possible using this high-resolution scanning method. Taphonomic observations such as orientation (oral side up, down, or oblique) of the fossils within the bed, posture and arrangement of ophiuroid arms and their relative spatial arrangement to one another could all be quantified *in situ*. In this Data Note, we also provide a brief step-by-step guide on creating, segmenting and ultimately combining sections of a fossiliferous layer containing abundant remains of delicate ophiuroids and stylophorans, in order to create a virtual view suited for quantitative and qualitative taphonomic analyses. An accompanying publication [21] presents the results of this analyses.

Material and methods

Excavation of the fossil bed

The initial discovery of the Karbonaatjies bed occurred during a preliminary study in 2014, when collected samples revealed rare, well-preserved individuals of an undescribed taxon of ophiuroid and stylophorans [22]. The study area is located within the Cape Fold Belt and the exposed rocks lithostratigraphically belong to the Lower Devonian (Emsian ~400 Ma) Voorstehoek Formation, Bokkeveld Group in South Africa [23]. The obrution bed was excavated from a road-cutting on Karbonaatjies farm, which is situated ~145 km northeast of Cape Town (GPS: 33°24003.600S, 19°52042.700E). A section of the obrution bed, approximately 2 x 1 m wide with an average thickness of 4 cm, was systematically excavated using a flat brick chisel, geological hammer and pickaxe. The highly-weathered nature of the outcrop caused the obrution bed to break up into 55 pieces during removal and each piece was labelled from A to WW (Fig. 1). All pieces were carefully reassembled in the lab and photographed.

Conventional palaeontological analysis of this deposit posed problems due to the fragile state of the fossils caused by the deep chemical weathering of the originally calcareous fossils, leaving only voids in the host rock, which is a silty, very fine-grained sandstone. For this reason, µCT scanning was used to analyses the Karbonaatjies obrution bed, which showed, among others that the sampled portion of this 2-3 cm thick layer contained over 1000 ophiuroid specimens of a new genus and species and hundreds of stylophorans.

Scanning, data processing and quality control

The μCT scanning was performed at the Stellenbosch University Central Analytical Facility with the aid of a walk-in microfocus X-ray CT scanner; the General Electric Phoenix V|Tome|X L24 model with additional NF180 option [24]. The CAF micro-CT instrument has a typical minimum voxel size of between 1 and 100 μm and can be used for samples that are up to 300 mm long and 200 mm wide. Samples were placed within a plastic bottle supported by dense polystyrene foam for scanning. Typical considerations for scan setup and parameter choices are outlined by du Plessis et al. [24]. A small wax ball was stuck to the upper surface of each sample in order to indicate right-way-up orientation as well as the relative position to other samples within the bed. To ensure that the X-ray spot size did not exceed the selected scan resolution, optimal X-ray scan parameters were chosen while using live digital X-ray images (e.g., for ideal X-ray penetration, we monitored the high transmitted brightness values). X-ray settings ranged for voltage from 160 to 240 kV (for larger samples) and for current from 200 to 220 μA depending on sample size, respectively. Detector shift was activated and background calibration was performed before each scan in order to minimize ring artefacts and achieve high image contrast. To reduce potential beam hardening artefacts, a 0.1 mm copper beam filter was used in all the scans. The samples in this study are relatively dense, rigid sandstone slabs with an average length of 200 mm. To obtain sharp images of larger samples, the voltage had to be raised up to 240 kV on the high-power tube allowing for more beam filtration (less beam hardening) and limiting the generation of other artefacts [25]. Scan time averaged from approximately 40-60 minutes depending on the size of the sample. Longer samples were scanned in sections (allowing for higher magnification). Using an exposure time of 500 ms per image, images were acquired in steps during a full 360° rotation. At each step position, the first image was discarded and the next two images averaged to obtain lower noise and sharper images. The acquired projection images (between 1400 and

2600 images per scan) were reconstructed using system-supplied Datos reconstruction software, where the choice of numbers of projections depends on sample size and magnification and was selected according to the guidelines in du Plessis et al. [24].

Digital analysis

The tomographic reconstruction dataset obtained from scanning was visualised and analysed using the VGStudioMax 3.1 software package (website: http://volumegraphics.com) to create a 3D view of individual fossils within each sample. This software was also used to produce images (e.g., screenshots) and animations.

Virtual preparation and dissection of the specimens involved a series of modified steps outlined by Abel [26] and Matthews [27]: (1) Density contrast enhancement: generation of a larger contrast between the grey scale values that represent the rock and surrounding air, by optimizing the grey value range on the histogram; (2) Register object: alignment of the sample to a specific coordinate system so that the top-down 2D viewer of the slices scrolls through the sample parallel to the bedding plane; (3) Surface determination: defining the material boundary of interest. This is generally the quickest and easiest way to separate a region of interest (ROI), however this was not possible because the fossils (preserved mostly as void space) have the same density or grey scale value as the permeating cracks in the samples and the surrounding air. It is for this reason that the region growing tool was predominantly used; (4) Region growing: generating a selection using a region growing algorithm (Fig. 2A). This is one of the simpler image segmentation methods used for 3D data, which essentially establishes the ROI (i.e., subparts of the volume data). The region growing tool allows the selection of a 'seed' point (in

this case the 'black' voxel of a fossil or the voids within the rock sample), the algorithm will expand the selection to all voxels connected to that seed point based on a defined tolerance of voxel grey values relative to the selected seed point. The threshold (selection of voxels with grey values within the selected grey value interval) changed from sample to sample but was generally around ±5000 in this study. Region growing was the most time-consuming step, as each individual fossil had to be segmented out in order to make the 3D volume rendering. Generally, when a fossil is scanned it is the minerals that make up the rock and the fossil itself that are compositionally differentiated so there is enough contrast to allow anatomical structures to be digitally visualised in 3D. This highlights one of the difficulties with CT scanning fossiliferous rock samples. If the compositional difference between the fossils and the host rock (matrix) is negligible, little or no information will be captured in the scans. However, the fossils of this study are predominantly mould fossils (i.e., 3-D imprints left behind in the host rock after the original organism was dissolved) with no internal mineralogical information, and this made segmentation much easier in that the contrast between the fossil (now air-filled void) and surrounding rock are very high. Finally, the last step involved (5) Volume rendering: generation of a 3D volume from the segmented 2D ROI's using a specific rendering algorithm in this case, the isosurface render. Isosurfaces are mathematically defined surfaces calculated from a volume along points of interest [3]. The dataset is treated as a volume comprising voxels (3D pixels that contain measurements of colour) instead of 2D pixels. Once the volume is created, the appearance of the volume objects can be manipulated (e.g., colour, transparency) to visualise the fossils in 3D (Fig. 2B). Lastly, once the fossils were segmented out, rendered in 3D and false-coloured accordingly, the fossiliferous rock samples were virtually 'stitched' together to recreate

a section of the obrution bed (Fig. 2C). This was achieved by using the volume import tool, each sample had to be manually aligned and placed in 3D space.

Data quality and limitations

In spite of the general preventative measures (e.g., using a copper filter, scanning perpendicular to the long axis of the sample), a number of the larger and longer samples scanned had artefacts that obscured details in the CT images. This makes interpretation and analysis very difficult, and sometimes even impossible (Fig. 3A, B). The major type of artefact identified is beam hardening, which is a problem that arises when a high energy polychromatic X-ray source is used to penetrate a dense sample. The strong absorption of the beam in a large sample causes low-energy photons to be absorbed more strongly that high-energy photons, resulting in unequal absorptions giving rise to this type of artefact. This problem often occurs in palaeontological samples because of the high density and large size of the fossil specimens and their matrix, resulting in low transmission and high noise [3]. In our study, it made identification and separation of the echinoderms arduous and even impossible in some cases, as more details towards the center of the samples were lost. To rectify this, many samples were cut into smaller sections and most of the non-fossiliferous host rock matrix was removed (Fig. 3C). These samples were subsequently re-scanned in sections to obtain higher magnification, especially for longer samples. Longer samples were scanned using a vertical multiscan procedure whereby different parts were scanned automatically with some overlap and the reconstruction software performed automatic stitching of multiple scans according to the accurate vertical translation distances used (no manual interface is necessary). However, due to the high power required, the X-ray source often became unstable causing errors and failed scans. For this reason, some individual parts of larger samples

were scanned separately and manually stitched. The advantage of this is that if there is a failure only one part needs to be rescanned, and less overlap is required for manual stitching, reducing the number of scans required for long or large objects. Finally, while all 55 samples were CT scanned, time and funding constraints allowed for only half of the bed (24 samples) to be rendered in 3D.

Potential uses

The dataset presented can be used as an example of how taphonomic and palaeoecological investigations can be conducted using μCT scanned rock samples within 3D space. Colourising the different taxa present in the virtually reconstructed fossiliferous bed played an important role in the taphonomic assessments. Ophiuroids were colour-coded according to their different orientations, light pink for oral (mouth) side up and dark pink for oral side down their presumed life position (Fig. 4A). Quantifying the percentage of ophiuroids in life position is a possible indication of the extent to which the ophiuroids may have been transported by storm-induced current and are subsequently reoriented before burial. Using the 3D reconstruction of the bed, most ophiuroids and stylophorans could be assigned to different taphonomic groups or decay stages based on their level of preservation. For example, ophiuroids that have fully articulated arms or stylophorans with a complete theca and aulacophore can be assigned to Group 1, which is intact or complete preservation. Other taphonomic categories were used for specimens in more fragmentary stages of preservation. Palaeoecological and taxonomic measurements such as specimen counts, ophiuroid disc or body diameter measured from the base of the arm to the opposite interradius, ophiuroid arm length measured in relation to the disc diameter, stylophoran

theca or body width and length, were all measured directly onto the 2D tomographic images using the digital caliper tool in VGStudioMax.

One of the unique perspectives of virtually viewing the obrution deposit in 3D is that it allows the examination of multiple levels of fossils preserved *in situ* within the bed (Fig. 4B). This is of particular interest because it provides insights into how the storm-generated sudden sediment supply smothered the echinoderms that lived on the Devonian seafloor. In this case, the dense assemblage of ophiuroids is arranged into vaguely laminated horizons with associated fossil shell debris. This helps understand the initial pre-burial storm conditions and the palaeoecologic fidelity of the resulting deposit. By setting the surrounding matrix to transparent in the program, other features such as the different arrangements of ophiuroid arms and the flexure of the stylophorans can be seen within the 3D space. One of the most striking features of the obrution bed was identified by using this technique. We observed that many of the ophiuroids had one or more arms extended upward into the overlying sediment. This has been identified as an escape posture and is comparable to modern examples of ophiuroids escaping from a sudden influx of sediment during storms [28]. Evidence of this behaviour in ancient ophiuroids is often difficult to interpret and is rarely observable in preserved ancient ophiuroids because traditional methods do not allow for such a comprehensive 3D view of the fossils [29, 30] (Fig 5).

The use of micro-CT scanning in palaeontological research has grown by leaps and bounds over the past 10 years. This study allowed the recognition of small, cryptic fossil taxa that would have been otherwise missed as well as the observation of key palaeontological features that are critical to the interpretation of the deposit.

Availability of supporting data

Data is available to download from the *GigaScience* GigaDB repository[31]. The presented dataset is available as two-dimensional X-ray projection images and stacks of reconstructed slice images for each sample scanned and can be viewed in any image viewer program. The final 3D render of the fossil bed is available as a VGL file which can be viewed on the free downloadable mvVGL program at https://www.volumegraphics.com/en/products/myvgl.html and is also available as a STL file that can be read by standard image viewer programs, e.g., ImageJ or FIJI. As the 3D surface-rendered image is very large (712MB), we have provided a version for web-based 3D visualisation in Sketchfab, embedded in the GigaDB entry and as Figure 5 in the paper. For 3D printing the STL model is also available in Thingiverse: https://www.thingiverse.com/thing:3233744

Abbreviations

3D: three dimensional; 2D: two dimensional; µCT: micro-computed tomography; CT: computed tomography: ROI: regions of interest.

Competing interests

The authors declare that they have no competing interests.

Authors' information

The dataset was acquired and compiled during the course of MR's MSc thesis at University of Cape Town supervised by EMB and WLT. MR, EMB, WLT conceived and designed the project, SLR and ADP prepared the data for upload and contributed to the technical side of this study.

MR wrote most of the final paper, and coordinated with co-authors (EMB, WLT, SLR, ADP) who performed critical revision of the manuscript.

Acknowledgements

We are grateful to Sandra Engels from Volume Graphics Gmbh for providing us with VGStudio Max at UCT. This project was supported by grants to WLT and EMB as well as postgraduate funding to MR from the DST-NRF Centre of Excellence in Palaeosciences (CoE in Palaeosciences). We gratefully acknowledge these financial contributions. Opinions expressed and conclusions arrived at are those of the authors and are not necessarily to be attributed to CoE PAL. We also gratefully acknowledge Chris Armit, Michael Bruce Meyer, Yu Liu for meaningfully contributing with their insightful comments to the overall quality of this study.

References

1. Sutton MD. Tomographic techniques for the study of exceptionally preserved fossils: Proceedings of the Royal Society. Series B. 2008; 275: 1587–1593. https://doi.org/10.1098/rspb.2008.0263
2. Cunningham JA, Rahman IA, Lautenschlager S, Rayfield EJ, Donoghue PCJ. A virtual world of paleontology: Trends in Ecology and Evolution. 2014; 29: 347–357. https://doi.org/10.1016/j.tree.2014.04.004
3. Sutton M, Rahman I, Garwood R. Techniques for virtual palaeontology. John Wiley & Sons; 2014 Feb 5.
4. Sutton MD, Rahman IA, Garwood RJ. Virtual paleontology—An overview: Paleontological Society Special Papers. 2017; 22:1–20.

5. Burrow CJ, Jones AS, Young GC. X-ray microtomography of 410 million-year-old optic capsules from placoderm fishes. Micron. 2005; 36(6): 551-557. https://doi.org/10.1016/j.micron.2005.05.005

6. Claessens LP, O'Connor PM, Unwin DM. Respiratory evolution facilitated the origin of pterosaur flight and aerial gigantism. PloS One: 2009; 4(2): e4497. https://doi.org/10.1371/journal.pone.0004497

7. Rossi M, Casali F, Romani D, Bondioli L, Macchiarelli R, Rook L. MicroCT Scan in paleobiology: application to the study of dental tissues. Nuclear Instruments and Methods in Physics Research Section B: Beam Interactions with Materials and Atoms. 2004; 747-750. https://doi.org/10.1016/S0168-583X(03)01697-5

8. Coates MI, Gess, RW, Finarelli JA, Criswell KE, Tietjen K. A symmoriiform chondrichthyan braincase and the origin of chimaeroid fishes. Nature. 2017; 541 (7636): 208-211. https://doi.org/10.1038/nature20806

9. Lautenschlager S. Fossils explained 69: From bone to pixel—fossil restoration and reconstruction with digital techniques. Geology Today. 2017; 33(4): 155-159. https://doi.org/10.1111/gto.12194

10. Stock SR, Veis A. Preliminary microfocus X-ray computed tomography survey of echinoid fossil microstructure. Geological Society Special Publication. 2003; 215: 225-235. https://doi.org/10.1144/GSL.SP.2003.215.01.21

11. Meyer M, Elliott D, Wood AD, Polys NF, Colbert M, Maisano JA, Vickers-Rich P, Hall M, Hoffman KH, Schneider G, Xiao S. Three-dimensional microCT analysis of the Ediacara fossil *Pteridinium simplex* sheds new light on its ecology and phylogenetic

affinity. Precambrian Research. 2014; 249: 79-87. https://doi.org/10.1016/j.precamres.2014.04.013

12. Lee, Sangmin, Shi GR, Park, Tae-Yoon S, Oh, Jae-Ryong, Mii, Horng-Sheng, Lee, Mirinae. Virtual palaeontology: the effects of mineral composition and texture of fossil shell and hosting rock on the quality of X-ray microtomography (XMT) outcomes using Palaeozoic brachiopods. Palaeontologia Electronica. 2017; 20.2.3T: 1-25.

13. Görög A, Szinger B, Tóth E, Viszkok J. Methodology of the micro-computer tomography on foraminifera. Palaeontologia Electronica. 2012; 15(1): 15.

14. Garwood RJ, Rahman IA, Sutton MD. From clergymen to computers – the advent of virtual palaeontology. Geology Today. 2010; 26: 96–100. https://doi.org/10.1111/j.1365-2451.2010.00753.x

15. Hamada T, Tateno S, Suzuki N. Three dimensional reconstruction of fossils with X-ray and computer graphics. Scientific Papers of the College of Arts and Sciences Univ. Tokyo. 1991; 41: 107–118.

16. Dominguez P, Jacobson AG, Jefferies RPS. Paired gill slits in a fossil with a calcite skeleton. Nature. 2002; 417: 841–844. https://doi.org/10.1038/nature00805

17. Garwood RJ, Dunlop JA. Morphology and systematics of Anthracomartidae (Arachnida: Trigonotarbida). Palaeontology. 2011; 54: 145–161. https://doi.org/10.1111/j.1475-4983.2010.01000.x

18. Donovan SK. The Processes of Fossilization. London; Belhaven Press. 1991; 303.

19. Brett CE, Moffat HA, Taylor WL. Echinoderm taphonomy, taphofacies, and lagerstätten. In: Waters, JA, Maples, CG (Eds.), Geobiology of Echinoderms. The Paleontological Society Papers. 1997; 3: 147–190.

20. Seilacher A, Reif WE, Westphal F. Sedimentological, ecological and temporal patterns of fossil-Lagerstätten. Philosophical Transactions of the Royal Society of London. 1985; 311: 5-23. https://doi.org/10.1098/rstb.1985.0134

21. Reid M. Taphonomy, paleoecology and taxonomy of an ophiuroid-stylophoran obrution deposit from the Lower Devonian Bokkeveld Group, South Africa. Thesis. 2017; 1-141. https://open.uct.ac.za/handle/11427/25404

22. Reid M, Bordy EM, Taylor W. Taphonomy and sedimentology of an echinoderm obrution bed in the Lower Devonian Voorstehoek Formation (Bokkeveld Group, Cape Supergroup) of South Africa. Journal of African Earth Sciences. 2015; 110: 135-149. https://doi.org/10.1016/j.jafrearsci.2015.04.009

23. Rust IC. The Evolution of the Paleozoic Cape Basin, Southern Margin of Africa. In: A.E.M. Nairn and F.G. Stehli (eds.), The Ocean Basins and Margins. 1: The South Atlantic, Plenum Publishing Corp, New York, U.S.A. 1973; 247-276. https://doi.org/10.1007/978-1-4684-3030-1_6

24. du Plessis, A, le Roux, SG, and Guelpa, A. The CT Scanner Facility at Stellenbosch University: an open access X-ray computed tomography laboratory. Nuclear Instruments and Methods in Physics Research Section B: Beam Interactions with Materials and Atoms. 2016; 384: 42-49.

25. Donovan SK. The Processes of Fossilization. London; Belhaven Press. 1991; 303.

26. Abel RL, Laurini CR, Richter M. A palaeobiologist's guide to 'virtual' micro-CT preparation. Palaeontologia Electronica. 2012; 15: 1-17.

27. Matthews T, du Plessis A. Using X-ray computed tomography analysis tools to compare the skeletal element morphology of fossil and modern frog (*Anura)* species. Palaeontologia Electronica. 2016; 19: 1-46.

28. Ishida Y, Fujita T. Escape behavior of epibenthic ophiuroids buried in the sediment: observations of extant and fossil *Ophiura sarsii sarsii*. In: Proceedings of the 10th International Echinoderm Conference. Dunedin 2000; 285-292.

29. Brett CE. Sedimentology, facies and depositional environments of the Rochester shale (Silurian, Wenlockian) in Western New York and Ontario. Journal of Sedimentary Petrology. 1983; 53(3): 947-972.

30. Jagt JWM. Late Cretaceous-Early Palaeogene echinoderms and the K/T boundary in the southeast Netherlands and northeast Belgium - Part 3: Ophiuroids. With a chapter on: Early Maastrichtian ophiuroids from Rügen (northeast Germany) and Møn (Denmark) by M Kutscher and JWM Jagt. Scripta Geologica. 2000; 121: 1-179.

31. Reid ML; Bordy EM; Taylor WL; le Roux SG; Du Plessis A (2018): Supporting data for "A micro X-ray computed tomography dataset of fossil echinoderms in an ancient obrution bed: a robust method for taphonomic and palaeoecologic analyses" GigaScience Database. http://dx.doi.org/10.5524/100539

Figure captions

Figure 1: Karbonaatjies obrution bed that was excavated from Karbonaatjies farm (~145 km northeast of Cape Town, South Africa). Each individual rock slab was given a reference letter in the field and subsequently reassembled in the lab.

Figure 2: A) Sample II, a long sample (26 cm by 12 cm) illustrating beam hardening artefacts causing the centre of the sample to appear to have darker voxels in the centre while the edges appear much brighter, even though the sample is homogeneous. B) Side view of Sample II showing how beam hardening artefact causes the pyrite minerals to give a ‘starburst’ appearance. C) Sample II after being cut and rescanned following the above-mentioned procedure.

Figure 3: A) Segmentation of ophiuroid specimens using the region growing tool in VGStudioMax. B) The resulting virtually reconstructed ophiuroids in 3D, rendered with colour and surrounding matrix set to transparent. C) Reconstruction of a portion of the fossil bed in 3D (shaded area in Figure 1). Black arrows point towards wax balls stuck to the upper surface of the samples to indicate right way up and relative positions.

Figure 4: A) Virtual reconstruction of samples SS, TT, TT2, TT3 and UU2 rendered with lights, colour and surrounding matrix set to 90% transparent. Approximately 80 articulated ophiuroids (light pink = ophiuroids oral side down; dark pink = ophiuroids oral side up); 13 paranacystids (green); one *Placocystella* (red) aulacophore fragment; numerous crinoid ossicles (blue); fragmented ophiuroid arms (pale orange) and large shell fragments (yellow) are all shown in 3D. B) Side view shows three vaguely defined ophiuroid ‘horizons’ as well as ophiuroid arms extended upward within the obrution bed.

Figure 5: As the 3D surface-rendered image is very large for convenient download and inspection we have provided a lower resolution version for web-based 3D visualisation in Sketchfab. This model was smoothed and decimated using Amira, with the modified version (1,392,960 faces, 49.6MB) uploaded to Sketchfab.

[Insert sketchfab window]

https://sketchfab.com/models/adad7e91e68049e9bc46f5250a33fd56

Figure 1 Click here to access/download;Figure;Figure 1.tif

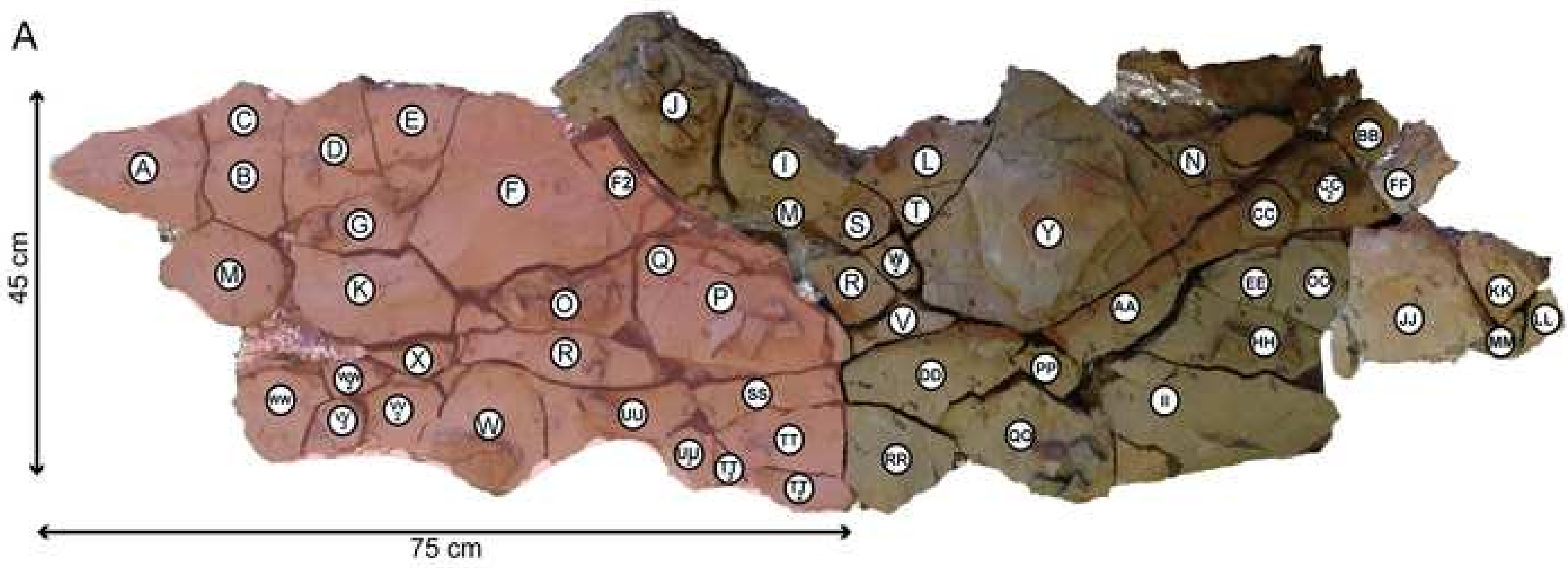

Figure 2

Click here to access/download;Figure;Figure 2.tif

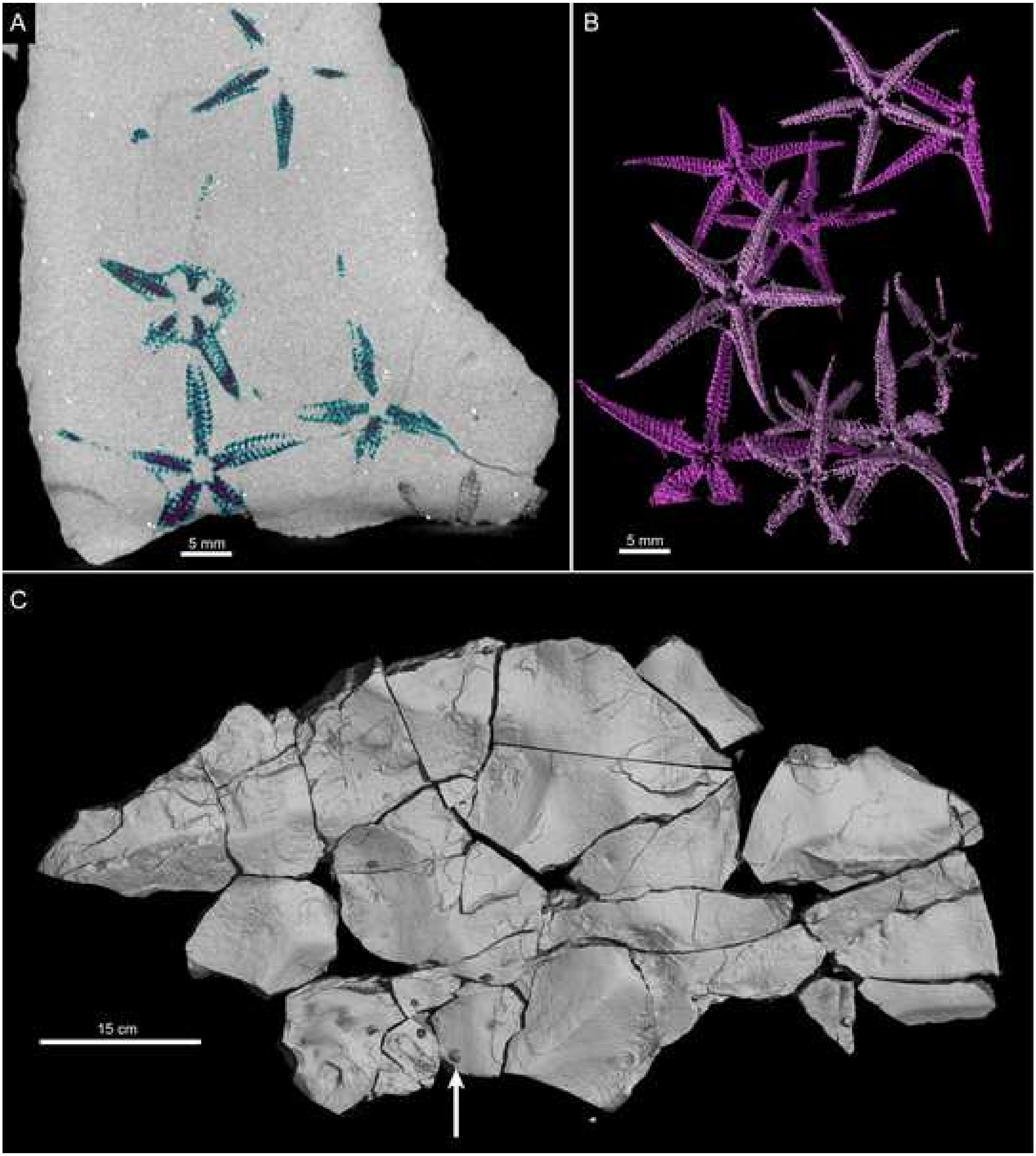

Figure 3

Click here to access/download;Figure;Figure 3.tif

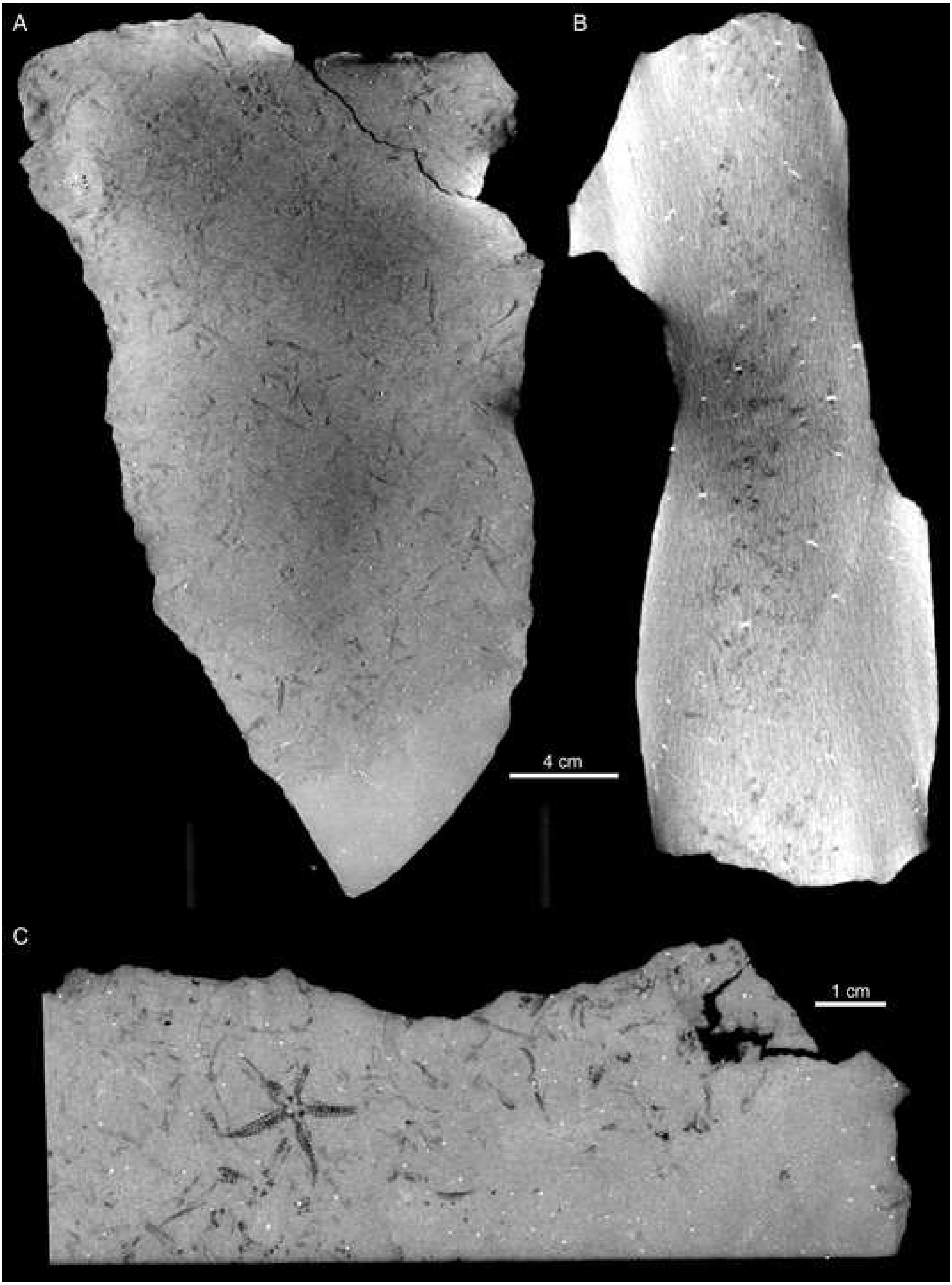

Figure 4 Click here to access/download;Figure;Figure 4.tif

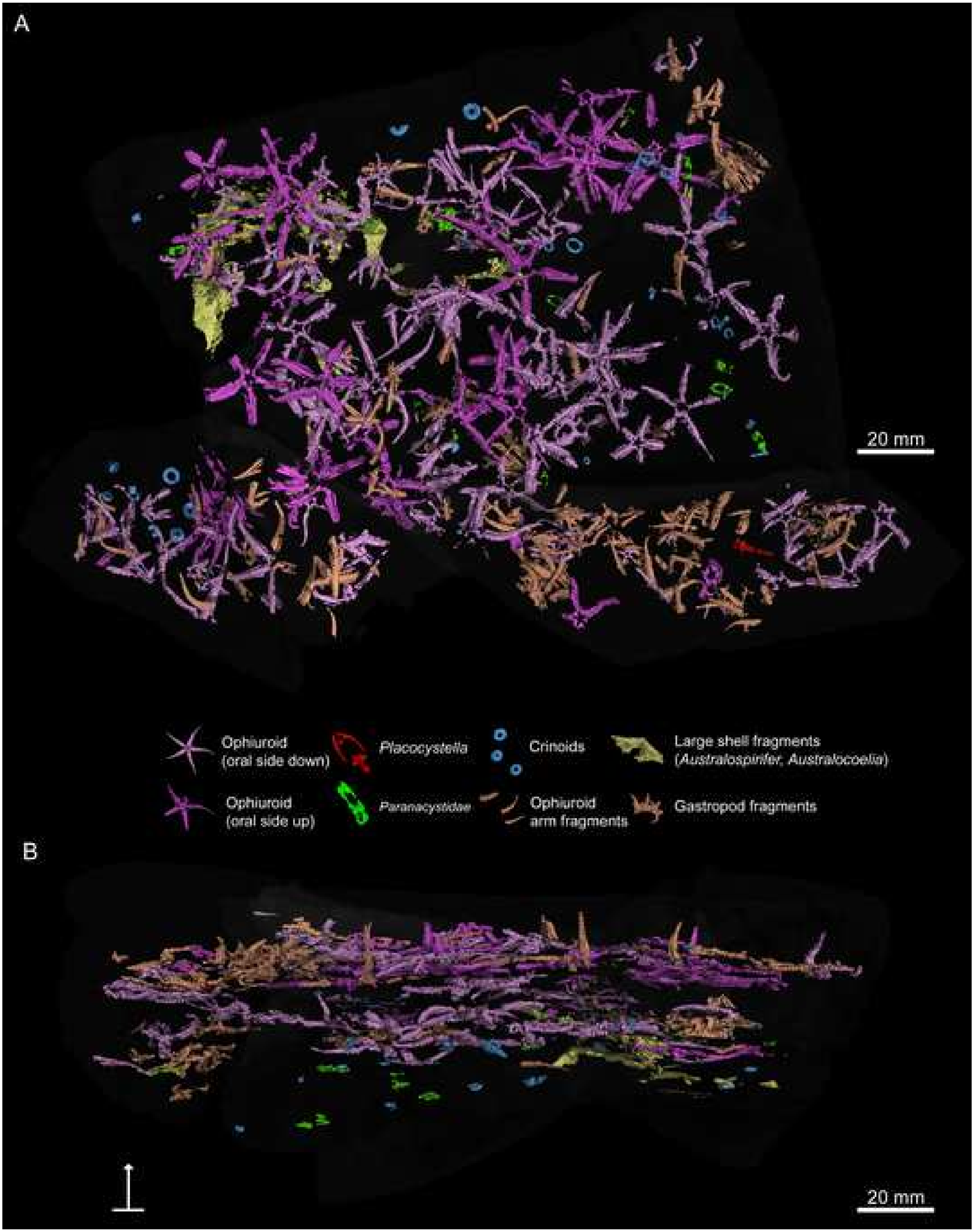

Figure 5. Thumbnail for Sketchfab

Click here to access/download;Figure;Screen Shot 2018-11-27 at 19.39.57.png

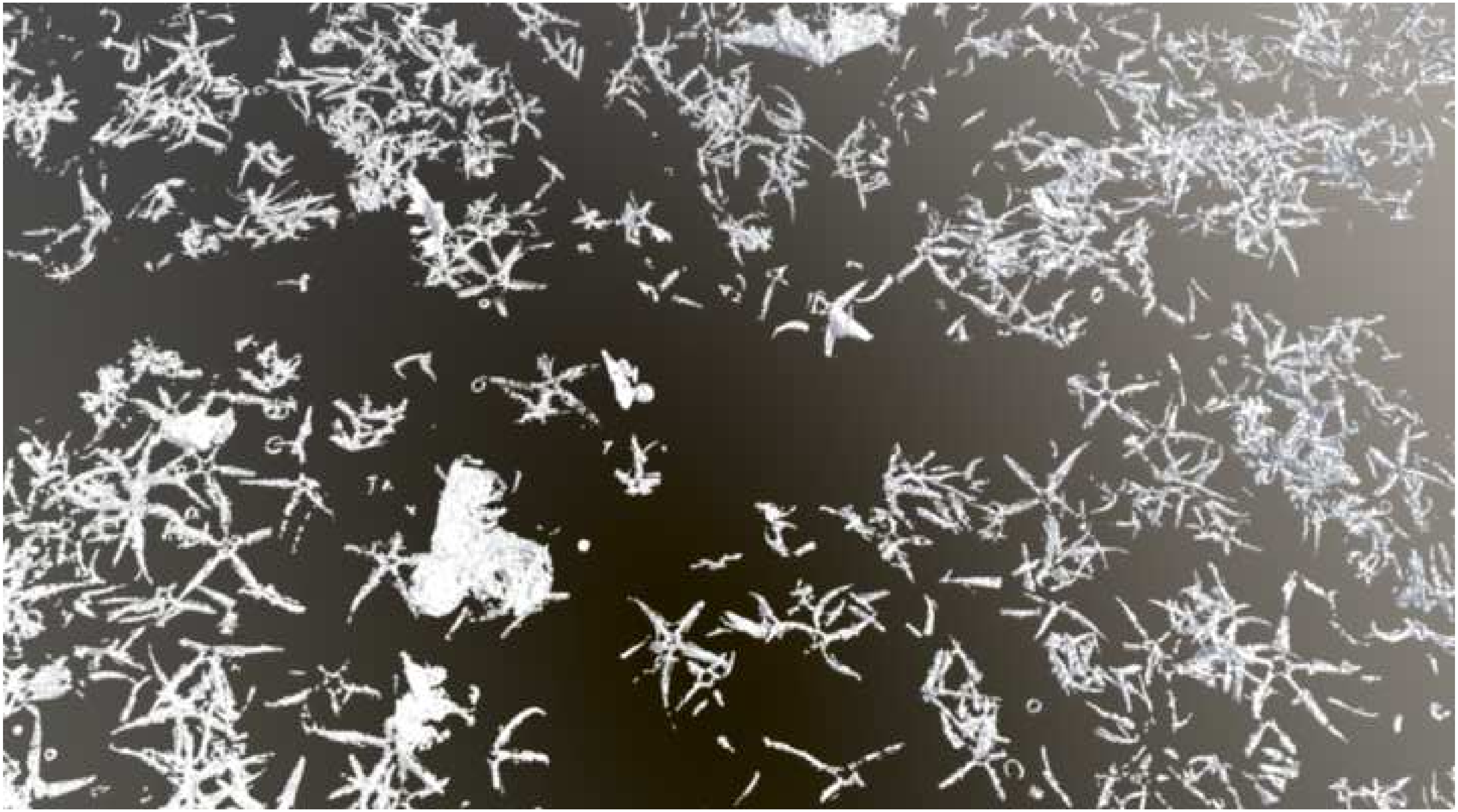

Personal Cover
Click here to access/download;Personal Cover;Letter.doc

DEPARTMENT OF GEOLOGICAL SCIENCES • e-mail: rdxmha001@myuct.ac.za
13 University Avenue, Upper Campus - University of Cape Town, Rondebosch, 7701, South Africa

October 16, 2018

Dear Editor,

Thank you very much for your helpful critical comments and for the support in improving this manuscript. We would like to resubmit the revised version of our manuscript entitled "A micro X-ray computed tomography dataset of fossil echinoderms in an ancient obrution bed: a robust method for taphonomic and palaeoecologic analyses". We have hopefully addressed the main concern of making the data more reusable by making an STL file of the entire fossil bed, which now can be viewed in many other commonly programs. We have uploaded the STL along with the revised manuscript. We also submitted Tiff stacks in the first instance that should be able to be viewed in Image J, please let us know if this is not the case.

Please feel free to contact me if you have any questions.

Yours truly,

Mhairi Reid
PhD student – Geology
http://www.geology.uct.ac.za/mhairi/reid/research
