# Supplementary material for: A micro X-ray computed tomography dataset of fossil echinoderms in an ancient obrution bed: a robust method for taphonomic and palaeoecologic analyses
Source: Gigascience. 2018 Dec 7;8(3):giy156. doi: 10.1093/gigascience/giy156 (PMC6505446; doi:10.1093/gigascience/giy156)
Supplement: Response_to_Reviewer_Comments_Original_Submission.pdf [file giy156_response_to_reviewer_comments_original_submission.pdf]

Author's Response To Reviewer Comments

Close

Responses to Reviewers

Reviewer #1: This is a novel and interesting study that utilises microcomputed tomography (micro-CT) to image complex echinoderm fossil specimens. The manuscript is well written, and the authors demonstrate quite beautifully how it is possible to use non-destructive 3D imaging techniques to segment starfish fossils from the host rock. Segmentations used in this study were accomplished using the commercial package VG Studio Max. The authors additionally provide an extremely useful step-by-step guide on how to generate these segmentations. From the figures, the 3D reconstructions look of sufficient quality for GigaDB to be able to generate surface reconstructions that can be interactively explored on the GigaDB website. Consequently, I suggest that GigaScience / GigaDB generate 3D models for this dataset to allow GigaDB users to interactively explore the echinoderm 3D reconstructions in the context of a web browser.

Response: We have now a STL file of the entire segmented fossil bed for this purpose. This process required creating meshes of all volumes and combining the meshes into one, using the "normal with simplification" meshing option.

Reviewer #1: Whereas the manuscript is well-written, the image data were submitted in proprietary formats that have some serious drawbacks in terms of accessibility and re-usability. The authors have submitted the volumetric image data in a proprietary image format that does not open with standard image viewers such as ImageJ or FIJI. I strongly encourage that the authors make the data available in a format that is more widely used (e.g. TIFF-stack) so that they are accessible (open, free, and universally implementable), and reusable (data are released with a clear and accessible data usage license). In this respect, I think it would be particularly useful if the authors make the image stacks available in a format that can be easily read by ImageJ or FIJI. Likewise, I suggest that the authors make the surface reconstruction file available in a more widely used format, such as OBJ or STL, rather than the proprietary VGL format that they have used. This will greatly encourage re-use of these data. Consequently, it is my recommendation that, in addition to VG Studio Max proprietary formats, the authors submit the supporting image data in more widely accessible formats.

Response: Thank you for the recommendation and we agree in making the image data more accessible. We submitted in VG Studio format which is possible to be accessed using their free viewer program, so is accessible to anyone who wishes, in windows or mac versions. The reason for this submission format is that the segmentation was performed in this software and the viewing of the segmented coloured data in ROIs is possible in this format. We also submitted Tiff stacks in the first instance so this should be accessible in imageJ, maybe the reviewer missed those? Finally, we now have made a STL of the entire segmented fossil bed. We hope it is now accessible and open to all!

Reviewer #2: Micro computed tomography (micro-CT) is by no means a novel technique in the field of Palaeontology. Well, you may be the first ones who apply this technique to the research of that Devonian assemblage from South Africa, but in the meantime, you basically follow the others' workflow published in the last years. I cannot see any innovation or

improvements in the "Methods" section. The dataset can be useful for the research of the assemblage, though. Therefore, I feel a journal in the field of Palaeontology is more suitable for this manuscript.

Response: While we agree that this not a novel technique, we feel that the application of the technique is different from the conventional approach. This is a Data Note paper demonstrating the largest combination of aligned microCT data to date to our knowledge, virtually recreating a fossil bed with large numbers of segmented features. We feel strongly it adds value, showing what is now possible with microCT and are very happy to have GigaScience reproduce our data so that everyone may have access to the digitized fossil bed. We currently have another paper in review in a paleontological journal on the taphonomy and paleoecology of the obrution bed illustrating the results of the microCT data obtained in detail.

Reviewer #3: I had reviewed a previous version of this manuscript and this is a much revised edition. A well put together MS!

Response: thank you, we appreciate the positive comment!

Close
