# Supplementary material for: A micro X-ray computed tomography dataset of fossil echinoderms in an ancient obrution bed: a robust method for taphonomic and palaeoecologic analyses
Source: Gigascience. 2018 Dec 7;8(3):giy156. doi: 10.1093/gigascience/giy156 (PMC6505446; doi:10.1093/gigascience/giy156)
Supplement: Reviewer_1_Report_1_(Original_Submission) -- Chris Armit — 9/19/2018 Reviewed [file giy156_reviewer_1_report_1_(original_submission).pdf]

**Reviewer Report**

**Title: A micro X-ray computed tomography dataset of fossil echinoderms in an ancient obrution bed: a robust method for taphonomic and palaeoecologic analyses**

**Version: Original Submission** **Date:** 9/19/2018

**Reviewer name: Chris Armit**

**Reviewer Comments to Author:**

This is a novel and interesting study that utilises microcomputed tomography (micro-CT) to image complex echinoderm fossil specimens. The manuscript is well written, and the authors demonstrate quite beautifully how it is possible to use non-destructive 3D imaging techniques to segment starfish fossils from the host rock. Segmentations used in this study were accomplished using the commercial package VG Studio Max. The authors additionally provide an extremely useful step-by-step guide on how to generate these segmentations.From the figures, the 3D reconstructions look of sufficient quality for GigaDB to be able to generate surface reconstructions that can be interactively explored on the GigaDB website. Consequently, I suggest that GigaScience / GigaDB generate 3D models for this dataset to allow GigaDB users to interactively explore the echinoderm 3D reconstructions in the context of a web browser.Whereas the manuscript is well-written, the image data were submitted in proprietary formats that have some serious drawbacks in terms of accessibility and re-usability.The authors have submitted the volumetric image data in a proprietary image format that does not open with standard image viewers such as ImageJ or FIJI. I strongly encourage that the authors make the data available in a format that is more widely used (e.g. TIFF-stack) so that they are accessible (open, free, and universally implementable), and reusable (data are released with a clear and accessible data usage license). In this respect, I think it would be particularly useful if the authors make the image stacks available in a format that can be easily read by ImageJ or FIJI. Likewise, I suggest that the authors make the surface reconstruction file available in a more widely used format, such as OBJ or STL, rather than the proprietary VGL format that they have used. This will greatly encourage re-use of these data.Consequently, it is my recommendation that, in addition to VG Studio Max proprietary formats, the authors submit the supporting image data in more widely accessible formats.

**Level of Interest**

Please indicate how interesting you found the manuscript: Choose an item.

**Quality of Written English**

Please indicate the quality of language in the manuscript: Choose an item.

**Declaration of Competing Interests**

Please complete a declaration of competing interests, considering the following questions:

- Have you in the past five years received reimbursements, fees, funding, or salary from an organisation that may in any way gain or lose financially from the publication of this manuscript, either now or in the future?

- Do you hold any stocks or shares in an organisation that may in any way gain or lose financially from the publication of this manuscript, either now or in the future?
- Do you hold or are you currently applying for any patents relating to the content of the manuscript?
- Have you received reimbursements, fees, funding, or salary from an organization that holds or has applied for patents relating to the content of the manuscript?
- Do you have any other financial competing interests?
- Do you have any non-financial competing interests in relation to this paper?

If you can answer no to all of the above, write 'I declare that I have no competing interests' below. If your reply is yes to any, please give details below.

I declare that I have no competing interests

I agree to the open peer review policy of the journal. I understand that my name will be included on my report to the authors and, if the manuscript is accepted for publication, my named report including any attachments I upload will be posted on the website along with the authors' responses. I agree for my report to be made available under an Open Access Creative Commons CC-BY license (http://creativecommons.org/licenses/by/4.0/). I understand that any comments which I do not wish to be included in my named report can be included as confidential comments to the editors, which will not be published.

I agree to the open peer review policy of the journal

To further support our reviewers, we have joined with Publons, where you can gain additional credit to further highlight your hard work (see: https://publons.com/journal/530/gigascience). On publication of this paper, your review will be automatically added to Publons, you can then choose whether or not to claim your Publons credit. I understand this statement. Yes
